# Interaction of Prions Causes Heritable Traits in *Saccharomyces cerevisiae*

**DOI:** 10.1371/journal.pgen.1006504

**Published:** 2016-12-27

**Authors:** Anton A. Nizhnikov, Tatyana A. Ryzhova, Kirill V. Volkov, Sergey P. Zadorsky, Julia V. Sopova, Sergey G. Inge-Vechtomov, Alexey P. Galkin

**Affiliations:** 1 St. Petersburg State University, Department of Genetics and Biotechnology, 199034 St. Petersburg, Russian Federation; 2 Vavilov Institute of General Genetics, St. Petersburg Branch, Russian Academy of Sciences, 199034 St. Petersburg, Russian Federation; 3 St. Petersburg State University, Research Park, Research Resource Center “Molecular and Cell Technologies”, St. Petersburg, Russian Federation; The University of Arizona, UNITED STATES

## Abstract

The concept of "protein-based inheritance" defines prions as epigenetic determinants that cause several heritable traits in eukaryotic microorganisms, such as *Saccharomyces cerevisiae* and *Podospora anserina*. Previously, we discovered a non-chromosomal factor, [*NSI*^+^], which possesses the main features of yeast prions, including cytoplasmic infectivity, reversible curability, dominance, and non-Mendelian inheritance in meiosis. This factor causes omnipotent suppression of nonsense mutations in strains of *S*. *cerevisiae* bearing a deleted or modified Sup35 N-terminal domain. In this work, we identified protein determinants of [*NSI*^+^] using an original method of proteomic screening for prions. The suppression of nonsense mutations in [*NSI*^+^] strains is determined by the interaction between [*SWI*^+^] and [*PIN*^+^] prions. Using genetic and biochemical methods, we showed that [*SWI*^+^] is the key determinant of this nonsense suppression, whereas [*PIN*^+^] does not cause nonsense suppression by itself but strongly enhances the effect of [*SWI*^+^]. We demonstrated that interaction of [*SWI*^+^] and [*PIN*^+^] causes inactivation of *SUP45* gene that leads to nonsense suppression. Our data show that prion interactions may cause heritable traits in *Saccharomyces cerevisiae*.

## Author Summary

The data presented in the paper deepens and enriches the concept of protein-based inheritance. According to this concept, prion conformational switches change protein functional activity, and such changes are inherited. Here, for the first time, we demonstrate that heritable traits may appear not only due to a conformational switch of one protein but also can be caused by interactions between different prions. To identify the novel epigenetic factor that causes suppression of nonsense mutations in yeast, we applied our original method of proteomic screening of prions. We have shown that two yeast proteins, which normally do not interact, in prion form demonstrate genetic interaction: one is the key determinant of the suppression of nonsense mutation, while the second enhances this effect. Thus, by analogy with monogenic and polygenic inheritance, in the framework of the prion concept, we can distinguish “monoprionic” and “polyprionic” inheritance. We assume that new examples of polyprionic inheritance will be revealed using modern proteomic methods for identification of prions.

## Introduction

Prions are proteins that convert between structurally distinct states, of which one or more is transmissible [1]. Prion formation leads to DNA-independent changes in heritable traits in microorganisms such as *Saccharomyces cerevisiae* and *Podospora anserina* [2,3]. For example, [*PSI*^+^] and [*ISP*^+^] prions, whose structural proteins are Sup35 and Sfp1, respectively, modulate nonsense suppression [2,4]; Swi1 in its prion state, [*SWI*^+^], causes a partial loss of function in utilizing non-glucose sugars and completely abolish yeast multicellularity [5,6], while Ure2 in its [*URE3*] form changes nitrogen catabolism [2]. The HET-s protein of *P*. *anserina* in prion state determine heterokaryon incompatibility corresponding to a cell death reaction, an event which occurs upon fusion of genetically distinct strains [7]. Thus, prion formation changes heritable information encoded at the protein level.

There are a number of works dedicated to the study of prion interactions, but currently we only know that pre-existing prions may facilitate the induction or elimination of other prions. It has been shown that pre-existing prions, such as [*PIN*^+^] or [*SWI*^+^], are required for induction but not for maintenance of [*PSI*^+^] [5,8–11]. Excluding Mod5 [12], all yeast prion proteins that form amyloid-like aggregates contain similar prion-forming regions rich in glutamine (Q) and/or asparagine (N) residues [13,14]. According to the cross-seeding model, pre-existing aggregates of one prion serve as the conformational template for newly forming prions [15]. Aggregates of Rnq1 or Swi1 in [*PIN*^+^] or [*SWI*^+^] strains, respectively, colocalize with overexpressing Sup35 only at the early stage of the initiation of [*PSI*^+^] prion formation, but at the latter stages the aggregates of these proteins do not colocalize [16]. Coexisting conformers of different prions do not physically interact probably because their conformations are spatially distinct. Some prions exhibit antagonistic relationships. For instance, the presence of [*URE3*] leads to the elimination of [*PSI*^+^] [17].

In this paper, we showed that coexisting prions may genetically interact, and this interaction causes heritable traits in *Saccharomyces cerevisiae*. Previously, we described [*NSI*^+^] (Nonsense Suppression Inducer) prion factor [18]. [*NSI*^+^] was shown to suppress the *ade1-14*_*UGA*_ and *trp1-289*_*UAG*_ nonsense alleles in background of modified Sup35 variants with decreased functional activity [18,19]. We demonstrated that the nonsense suppressor phenotype of [*NSI*^+^] cells, i.e., their growth on–Ade or–Trp synthetic media, is caused by defects in translation termination [19,20]. This factor does not depend on [*PSI*^+^] prion, because [*NSI*^+^] phenotype is manifested in the strains containing deletion of N-terminal prion-forming domain of Sup35.” Additionally, [*NSI*^+^] cells exhibit growth defects on media containing galactose or glycerol as the sole carbon source [19]. Like known yeast prions, [*NSI*^+^] shows reversible curability, non-Mendelian inheritance, and cytoplasmic infectivity. Deletion of the chaperone Hsp104 or its inactivation by Guanidine-Hydrochloride (GuHCl) causes elimination of [*NSI*^+^] [18]. We previously used large-scale overexpression screens to reveal the genes affecting [*NSI*^+^] manifestation, but we did not identify the structural gene of this factor [19,21]. Recently we developed the proteomic method for identification of yeast prion proteins that form amyloid-like aggregates resistant to treatment with ionic detergents [22]. Here, using this method we demonstrated that the nonsense suppression in the [*NSI*^+^] strain is a result of interaction between [*PIN*^+^] and [*SWI*^+^] prions. We showed that prion inactivation of Swi1 protein decreases the expression of the translation termination factor eRF1, and [*PIN*^+^] enhances this effect.

## Results

### [*NSI*^+^] strain contains [*PIN*^+^] prion which acts as the enhancer of the nonsense suppression

**Fig 1:**
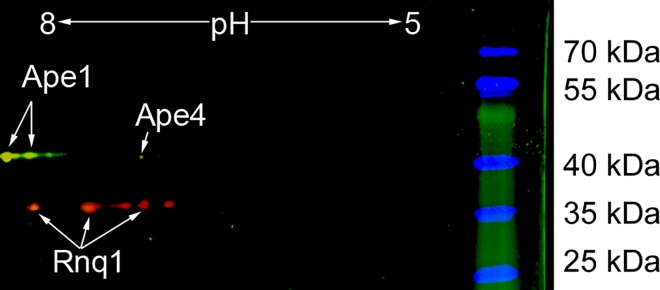
2D-DIGE image of proteins forming SDS-insoluble aggregates isolated from the 1-1-D931 [*NSI*^+^] and 1-1-1-D931 [*nsi*^-^] strains. Spots corresponding to proteins from 1-1-D931 [*NSI*^+^] cells are pseudocolored in red (Cy5), while proteins from the 1-1-1-D931 [*nsi*^-^] are pseudocolored in green (Cy3). Yellow spots correspond to proteins present in both samples. A strip with a pH gradient of 5–8 was used. Proteins identified by mass-spectrometry are indicated. The mass spectra of identified proteins are listed in S1–S3 Figs.

To identify the proteins whose prion conversion determines the [*NSI*^+^] phenotype, we used a method for proteomic screening and identification of amyloid proteins (PSIA) which we had previously developed and successfully applied for identification of yeast prions [22]. This method is based on the resistance of prion aggregates to treatment with ionic detergents such as sodium dodecyl sulfate (SDS). To reveal prion proteins that determine the manifestation of the [*NSI*^+^] factor, we carried out a comparative analysis of proteins forming SDS-resistant aggregates in the 1-1-D931 [*NSI*^+^] and 1-1-1-D931 [*nsi*^-^] isogenic strains. Proteins forming aggregates resistant to 1% SDS were solubilized, labeled with Cy5 ([*NSI*^+^]) and Cy3 ([*nsi*^-^]) fluorescent dyes and analyzed by two-dimensional gel electrophoresis (2D-DIGE) (Fig 1). Proteins present only in the test sample ([*NSI*^+^]) are pseudocolored in red, proteins present only in the control sample ([*nsi*^-^]) are pseudocolored in green and yellow spots correspond to proteins that were present in both samples. Such yellow spots were identified as the aminopeptidases Ape1 and Ape4 (Fig 1, S1 and S2 Figs) that we had detected in our previous study in different yeast strains [22]. Red spots specific to the [*NSI*^+^] sample (Fig 1) were identified as Rnq1 (S3 Fig), which is the structural protein of [*PIN*^+^] prion [10,23].

**Fig 2:**
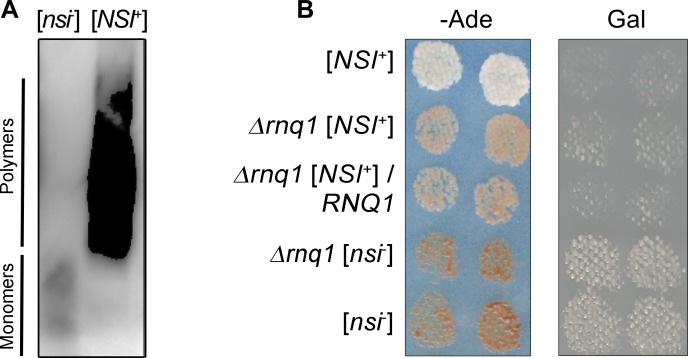
[*NSI*^+^] strain contains [*PIN*^+^] prion which acts as the enhancer of the nonsense suppression. (A) SDD-AGE of protein lysates extracted from the 1-1-D931 [*NSI*^+^] and 1-1-1-D931 [*nsi*^-^] strains expressing pCUP1-RNQ1-CFP(LEU2) plasmid. Protein lysates were treated with 1% SDS at room temperature. SDS-resistant aggregates of Rnq1-CFP were detected using monoclonal rabbit primary antibodies against GFP [E385] (ab32146) (Abcam, Great Britain) and ECL Prime Western Blotting Detection Reagent kit (GE Healthcare, USA). (B) The effects of *RNQ1* deletion on the [*NSI*^*+*^] phenotypic manifestation. *RNQ1* deletion was obtained as described in Materials and Methods. The [*nsi*^-^] and [*nsi*^-^] *rnq*Δ strains were obtained from the corresponding [*NSI*^+^] strains by GuHCl treatment. To express *RNQ1*, the 5-1-1-D931 [*NSI*^+^] *rnq*Δ strain and its [*nsi*^-^] derivative were transformed with the YGPM25a02 plasmid containing a genomic fragment encoding *RNQ1* under the control of its endogenous promoter. Other strains presented in this Figure were transformed with an empty vector expressing only the *LEU2* gene. Transformants were selected on–Leu medium with 150 μM CuSO_4_ and replica-plated on–Leu–Ade medium or–Leu medium with 150 μM CuSO_4_ containing galactose as the sole carbon source. Images were taken after 5 days of incubation of–Ade plates or after 3 passages on Gal plates.

To confirm the presence of [*PIN*^+^] in the [*NSI*^+^] strain, we transformed 1-1-D931 [*NSI*^+^] and 1-1-1-D931 [*nsi*^-^] cells with pCUP1-RNQ1-CFP(LEU2) plasmid and grew the cells for 48 h at 30°C in liquid–Leu selective medium containing 150 μM CuSO_4_. Next, we analyzed Rnq1-CFP aggregation in these strains using semi-denaturing detergent agarose gel electrophoresis (SDD-AGE) assay (Materials and Methods). Rnq1-CFP formed SDS-resistant oligomers in the [*NSI*^+^] strain but not in [*nsi*^-^] (Fig 2A). Thus, we confirmed that the [*NSI*^+^] strain bears the [*PIN*^+^] prion.

Next, we tested whether [*PIN*^+^] affects the phenotypic manifestation or maintenance of the [*NSI*^+^] factor. We obtained three independent clones containing PCR-generated deletion of *RNQ1* in the 1-1-D931 [*NSI*^+^] strain (Materials and Methods) and analyzed the phenotypic manifestation of the resulting strain 5-1-1-D931 [*NSI*^+^] *rnq1*Δ. All clones containing deletion of *RNQ1* were characterized by a decreased level of nonsense suppression (Fig 2B) which was intermediate between those of [*NSI*^+^] and [*nsi*^-^]. However, deletion of *RNQ1* did not affect cell growth on media containing galactose as the sole carbon source (Fig 2B). The weak suppressor phenotype of 5-1-1-D931 [*NSI*^+^] *rnq*Δ was stably inherited in mitotic progeny, and reintroduction of the *RNQ1* gene on the plasmid did not restore strong nonsense suppression (Fig 2B). Moreover, the weak suppressor phenotype of 5-1-1-D931 [*NSI*^+^] *rnq*Δ strain was completely eliminated by curing on YPD medium containing 5 mM Guanidine Hydrochloride (GuHCl) (Fig 2B). These data suggest that the strong nonsense suppressor phenotype of the [*NSI*^+^] strains is a result of interaction between [*PIN*^+^], which acts as the enhancer of nonsense suppression, and a second, unknown prion.

### [*SWI*^+^] is the key determinant of the nonsense suppression in the [*NSI*^+^] strain

Since we proposed that weak nonsense suppression in the [*NSI*^+^] *rnq*Δ strain was caused by an unknown prion that was undetectable by the standard PSIA approach, we modified the PSIA protocol to improve its sensitivity. Although 2D-DIGE is a useful comparative method, it has strong limitations. For example, minor proteins cannot be detected on the gel, and proteins with extreme pI typically do not enter the gel. In addition, not all amyloids are soluble in UTC (8 M urea, 2 M thiourea, 4% CHAPS, and 30 mM TrisHCl pH 8.5) buffer [22]. In this study, we used a novel variant of PSIA called PSIA-LC-MALDI. This method consists of (i) the previously described procedure of isolation of detergent-resistant protein fractions [22] followed by (ii) solubilization of proteins with formic acid and by boiling in SDS-PAGE loading buffer, (iii) purification of proteins from detergent, trypsinization and (iv) separation of tryptic peptides by high-performance liquid chromatography coupled with matrix-assisted laser desorption/ionization mass spectrometry (LC-MALDI). For a detailed description of PSIA-LC-MALDI, see Materials and Methods. We applied this method to identify detergent-resistant proteins from the 4-1-1-D931 [*NSI*^+^] and 1-4-1-1-D931 [*nsi*^-^] strains. Yeast core ribosomal proteins that were presented in SDS-resistant fraction were excluded from this table because they form SDS-resistant non-amyloid complexes [24]. From this analysis we identified 46 proteins with Mascot score >60 at a significance level of p<0.05; 42 of them were presented in SDS-resistant fraction of both, [*NSI*^+^] and [*nsi*^-^] strains, while 4 were identified in the [*NSI*^+^] strain only: Rnq1, Swi1, Mit1, and Sis1. (S1 Table; MS/MS spectra of Rnq1, Swi1, Sis1, and Mit1 proteins are presented in S4–S7 Figs, respectively).

The presence of Rnq1 in the SDS-resistant fraction of the [*NSI*^+^] strain supports our conclusion that the [*NSI*^+^] strain contains the [*PIN*^+^] prion. Swi1 is the structural protein of the [*SWI*^+^] prion [5], whereas Mit1 is an evolutionally conserved transcriptional regulator of pseudohyphal growth [25] whose amino acid sequence contains an extremely asparagine-rich region. The presence of Sis1 chaperone in the SDS-resistant fraction of the [*NSI*^+^] strain is not surprising, because Sis1 binds [*PIN*^+^] aggregates [26] and is important for its propagation [27].

**Fig 3:**
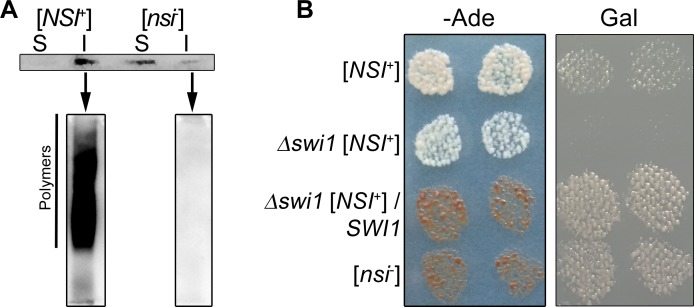
[*SWI*^+^] prion is a key determinant of nonsense suppression in [*NSI*^+^] strains. (A) Sedimentation analysis of Swi1(1–297)-YFP protein from the 4-1-1-D931 [*NSI*^+^] and 1-4-1-1-D931 [*nsi*^-^] strains expressing pCUP1-SWI1(1–297)-YFP (URA3) plasmid. Soluble (S) and insoluble (I) fractions were obtained as indicated in Materials and Methods. Swi1(1–297)-YFP was detected using monoclonal rabbit primary antibodies against GFP [E385] (ab32146) (Abcam, Great Britain) and ECL Prime Western Blotting Detection Reagent kit (GE Healthcare, USA). Next, SDD-AGE analysis of insoluble fractions of [*NSI*^+^] and [*nsi*^-^] strains comprising Swi1(1–297)-YFP was performed. (B) The effects of *SWI1* deletion on the [*NSI*^*+*^] phenotypic manifestation. *SWI1* deletion was obtained as described in Materials and Methods. To express *SWI1*, the 11-1-1-D931 [*NSI*^+^] *swi1*Δ strain was transformed with the YGPM19p21 plasmid from the YSC4613 genomic library, containing a genomic fragment encoding *SWI1* under the control of its endogenous promoter. Other strains presented in this Figure were transformed with an empty vector expressing only the *LEU2* gene. Transformants were selected on–Leu medium with 150 μM CuSO_4_ and replica-plated on–Leu–Ade or–Leu Gal media with 150 μM CuSO_4_. Images were taken after 5 days of incubation of–Ade plates or after 3 passages on Gal plates.

To analyze whether the [*NSI*^+^] strain contains Swi1 in the [*SWI*^+^] state, the [*NSI*^+^] and [*nsi*^-^] strains were transformed with pCUP1-SWI1(1–297)-YFP(URA3) plasmid, cell lysates were separated by centrifugation onto the soluble (S) and insoluble (I) fractions and Swi1(1–297)-YFP protein was detected by Western blotting. The Swi1(1–297)-YFP protein in the [*NSI*^+^] strain was detected in the insoluble fraction only, whereas in the [*nsi*^-^] strain it was presented mostly in the soluble fraction (Fig 3A). Next, the insoluble fractions from the [*NSI*^+^] and [*nsi*^-^] strains were analyzed by semi-denaturing detergent agarose gel electrophoresis (SDD-AGE). We showed that Swi1(1–297)-YFP forms SDS-resistant polymers in the [*NSI*^+^] strain but not in [*nsi*^-^] (Fig 3A). These data support the results of PSIA-LC-MALDI and confirm that the [*NSI*^+^] strain contains Swi1 in the [*SWI*^+^] state.

We demonstrated that the PCR-generated deletion of *SWI1* leads to very strong nonsense suppression and an almost complete absence of growth on medium containing galactose as the sole carbon source (Fig 3B). To analyze the effect of [*SWI*^+^] elimination, we re-introduced *SWI1* by transformation of the 11-1-1-D931 *swi1*Δ strain with the YSC4613 genomic library (Open Biosystems, USA) plasmid containing the *SWI1* gene. Next, we analyzed nonsense suppression and growth of the transformants on media without adenine or with galactose as the sole carbon source. Cells that lost the [*SWI*^+^] prion had a phenotypic manifestation identical to [*nsi*^-^] (Fig 3B). Probably, [*SWI*^+^] is the key determinant of the [*NSI*^+^] factor that regulates both nonsense suppression and growth on the medium containing galactose as the sole carbon source. However, considering that the strong suppressor phenotype manifested only in strains containing both [*SWI*^+^] and [*PIN*^+^] prions, we can assume that the manifestation of the [*NSI*^+^] factor results from interaction between [*SWI*^+^] and [*PIN*^+^] prions.

### Mit1 forms detergent-resistant aggregates independently of the [*NSI*] status of the cells

**Fig 4:**
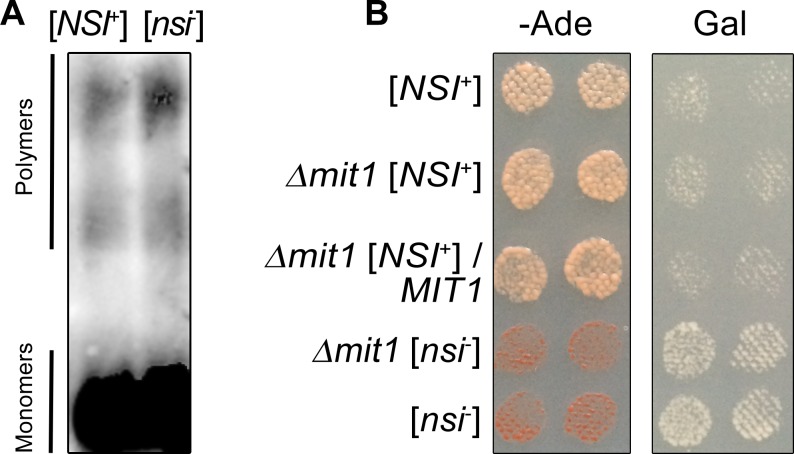
Mit1 is not a determinant of the [*NSI*^+^] factor. (A) SDD-AGE assay of protein lysates extracted from the 4-1-1-D931 [*NSI*^+^] and 1-4-1-1-D931 [*nsi*^-^] strains expressing pMIT1-MIT1-GFP(URA3) plasmid. Cells were grown for 48 h at 30°C in liquid–Ura selective medium containing 150 μM CuSO_4_. Protein lysates were treated with 1% SDS at room temperature. Mit1-GFP was detected with monoclonal rabbit primary antibodies against GFP [E385] (ab32146) (Abcam, Great Britain) and ECL Prime Western Blotting Detection Reagent kit (GE Healthcare, USA). (B) *MIT1* deletion does not affect the [*NSI*^*+*^] phenotypic manifestation. *MIT1* deletion was obtained as described in Materials and Methods. The [*nsi*^-^] derivative of the 2–936 [*NSI*^+^] *mit1*Δ was obtained by GuHCl treatment. To express *MIT1*, 2–936 [*NSI*^+^] *mit1*Δ strain was transformed with YGPM21o12 plasmid from the YSC4613 genomic library containing a genomic fragment encoding *MIT1* under the control of its endogenous promoter. Other strains presented in this Figure were transformed with a vector expressing only the *LEU2* gene. Transformants were selected on–Leu medium with 150 μM CuSO_4_ and replica-plated on–Leu–Ade or–Leu Gal media with 150 μM CuSO_4_. Images were taken after 5 days of incubation of–Ade plates or after 3 passages on Gal plates.

The third asparagine-glutamine-rich protein identified only in SDS-resistant aggregate fraction of the [*NSI*^+^] strain was Mit1. We analyzed whether Mit1 is present in the aggregated state in the [*NSI*^+^] strain by SDD-AGE. The 4-1-1-D931 [*NSI*^+^] and 1-4-1-1-D931 [*nsi*^-^] strains were transformed with a pMIT1-MIT1-GFP(URA3) centromeric plasmid that expresses the Mit1 protein fused with GFP under the control of its endogenous promoter. Interestingly, the results of SDD-AGE demonstrated that a small portion of Mit1-GFP protein formed detergent-resistant aggregates in both [*NSI*^+^] and [*nsi*^-^] strains (Fig 4A). Considering that the level of Mit1-GFP expression in our experiment was close to physiological, we propose that some portion of this protein permanently forms amyloid-like aggregates in yeast cells.

Further, we analyzed the influence of *MIT1* deletion on the phenotypic manifestation of the [*NSI*^+^] strain. The levels of nonsense suppression and growth on galactose-containing medium were identical between the 2–936 [*NSI*^+^] *mit1*Δ strain and 1-1-D931 [*NSI*^+^] strain (Fig 4B). Additionally, expression of *MIT1* on the plasmid in the 2–936 [*NSI*^+^] *mit1*Δ strain did not affect the [*NSI*^+^] phenotype (Fig 4B). Together, these results indicate that Mit1 forms SDS-resistant aggregates independently of the [*NSI*] status of the cell and does not affect the maintenance or phenotypic manifestation of [*NSI*^+^].

### [*SWI*^+^] and [*PIN*^+^] show genetic interaction similar to complementary interaction of classical genes

**Fig 5:**
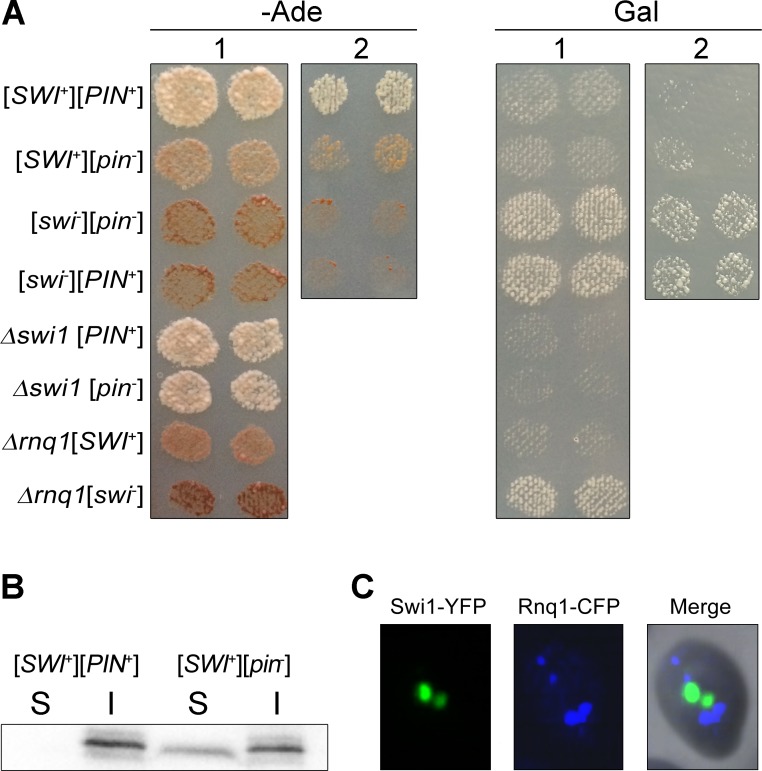
[*SWI*^+^] and [*PIN*^+^] prions demonstrate complementary interaction. (A) Comparative analysis of the growth of strains containing combinations of [*prion*^*-*^] or [*PRION*^+^] states for Rnq1 and Swi1 as well as deletions of the corresponding genes. “1”–The [*SWI*^+^][*pin*^-^] and [*swi*^-^][*PIN*^+^] strains were obtained from the 1-1-D931 [*SWI*^+^][*PIN*^+^] strain by deletion with subsequent reintroduction of *RNQ1* and *SWI1* genes, respectively (see “Materials and Methods”). The [*swi*^-^][*pin*^-^] strain was obtained from the 1-1-D931 [*SWI*^+^][*PIN*^+^] strain by GuHCl curing. “2”–The 26-1-4-1-1-D931 [*swi*^-^][*PIN*^+^], 12-1-4-1-1-D931 [*SWI*^+^][*pin*^-^], and 16-1-4-1-1-D931 [*SWI*^+^][*PIN*^+^] strains were obtained by transformation of the 1-4-1-1-D931 [*swi*^-^][*pin*^-^] recipient yeast cells with the 1-1-D931 [*SWI*^+^][*PIN*^+^] protein lysates followed by analysis of [*SWI*] and [*PIN*] status of the cells as described in “Materials and Methods”. Images were obtained after 5 days of incubation on–Ade plates with 150 μM CuSO_4_ or after 3 passages on Gal plates. (B) Sedimentation analysis of Swi1(1–297)-YFP protein from the 16-1-4-1-1-D931 [*SWI*^+^][*PIN*^+^] and 12-1-4-1-1-D931 [*SWI*^+^][*pin*^-^] strains expressing the pCUP1-SWI1(1–297)-YFP (URA3) plasmid. Soluble (S) and insoluble (I) fractions were obtained as indicated in Materials and Methods. Swi1(1–297)-YFP was detected using monoclonal rabbit primary antibodies against GFP [E385] (ab32146) (Abcam, Great Britain) and ECL Prime Western Blotting Detection Reagent kit (GE Healthcare, USA). (C) Analysis of the colocalization of Swi1-YFP and Rnq1-CFP aggregates. The cells of the D938 [*SWI*^+^][*PIN*^+^] strain were co-transformed with p426GPD–SWI1YFP and pCUP1-RNQ1-CFP(LEU2) plasmids. Transformants were selected on–Ura–Leu selective media with 150 μM CuSO_4_ and incubated for 48 h prior to fluorescence microscopy.

Once we demonstrated that the [*NSI*^+^] phenotype is determined by two prions, [*SWI*^+^] and [*PIN*^+^], we decided to analyze their interactions. First, we compared the growth of strains containing different combinations of [*SWI*^+^] and [*PIN*^+^] prions as well as deletions of the *SWI1* or *RNQ1* genes. The phenotypes of all these strains were analyzed on medium without adenine and on medium containing galactose as the sole carbon source (Fig 5A). As shown in the figure, [*swi*^-^][*pin*^-^] and [*swi*^-^][*PIN*^-+^] strains did not grow on–Ade medium. [*SWI*^+^] caused a weak nonsense suppression in the absence of [*PIN*^-+^]. Comparative analysis of the growth of the [*SWI*^+^][*PIN*^+^] and [*SWI*^+^][*pin*^-^] strains on–Ade medium showed that [*PIN*^-+^] enhanced [*SWI*^+^]-dependent nonsense suppression. The strongest suppressor phenotype was observed on the background of *SWI1* gene deletion in three independently obtained clones, and in this case suppression did not depend on [*PIN*^+^] (Fig 5A). These data suggest that suppression of nonsense mutation depends on Swi1 inactivation. Prion inactivation of Swi1 protein causes weak nonsense suppression, [*PIN*^+^] enhances this effect, whereas deletion of *SWI1* leads to strongest nonsense suppressor phenotype. To reproduce these results, the strains with different combination of prions (26-1-4-1-1-D931 [*swi*^-^][*PIN*^+^], 12-1-4-1-1-D931 [*SWI*^+^][*pin*^-^], and 16-1-4-1-1-D931 [*SWI*^+^][*PIN*^+^]) were obtained by transformation of [*swi*^-^][*pin*^-^] cells with protein lysates (see “Materials and Methods”). The recipient 1-4-1-1-D931 [*swi*^-^][*pin*^-^] strain was transformed with protein extract from the 1-1-D931 [*SWI*^+^][*PIN*^+^] strain and with plasmid pRNQ1-GFP (URA3). The clones that acquired strong and weak suppressor phenotypes were selected. All these clones lost nonsense suppressor phenotype after GuHCl treatment. The [*swi*^-^][*PIN*^+^] protein transformants which had the same phenotype as the recipient [*swi*^-^][*pin*^-^] cells, but contained Rnq1-GFP aggregates, were selected by fluorescent microscopy (S8 Fig). The [*SWI*^+^] status of cells that acquired strong or weak suppressor phenotype was confirmed by analysis of Swi1(1–297)-YFP fluorescent aggregates (S9 Fig). Thus, we obtained the [*SWI*^+^][*PIN*^+^], [*SWI*^+^][*pin*^-^], and [*swi*^-^][*PIN*^+^] strains by two different methods and showed that suppression of *ade1-14*_*UGA*_ nonsense mutation depends on interaction of [*SWI*^+^] and [*PIN*^+^] prions (Fig 5A). Such interaction of two prions is very similar to the classical complementary interaction of two genes, where dominant allele of one gene enhances the manifestation of dominant allele of other gene. The second analyzed phenotype, i.e., growth on medium containing galactose as the sole carbon source, was determined by [*SWI*^+^] only, whereas the presence or absence of [*PIN*^+^] did not affect this phenotypic trait (Fig 5A).

Since nonsense suppression in [*SWI*^+^] strains depends on [*PIN*] status, we proposed that [*PIN*^+^] might affect aggregation of Swi1 protein in prion form. To test this hypothesis, we compared the levels of Swi1 aggregates in the 12-1-4-1-1-D931 [*SWI*^+^][*pin*^-^] and 16-1-4-1-1-D931 [*SWI*^+^][*PIN*^+^] strains by centrifugation analysis. The data presented in Fig 5B demonstrate that Swi1(1–297)-YFP protein is detected in the [*SWI*^+^][*PIN*^+^] cells in the insoluble fraction only, whereas in the [*SWI*^+^][*pin*^-^] cells Swi1(1–297)-YFP is presented in both, soluble and insoluble fractions. These data show that [*PIN*^+^] directly or indirectly enhances Swi1 aggregation in the [*SWI*^+^] strains.

Recently, it was shown that coexisting aggregates of Swi1-YFP and Rnq1-CFP do not colocalize in [*PIN*^+^][*SWI*^+^] cells [16]. We also demonstrated that Swi1-YFP and Rnq1-CFP proteins do not colocalize in the D938 [*SWI*^+^][*PIN*^+^] strain (Fig 5C). Thus, the presence of [*PIN*^+^] enhances formation of Swi1-YFP aggregates in [*SWI*^+^] strains, but this effect is not mediated by a physical interaction between Rnq1 and Swi1 proteins.

### [*SWI*^*+*^] and [*PIN*^*+*^] prions affect the level of *SUP45* mRNA

**Fig 6:**
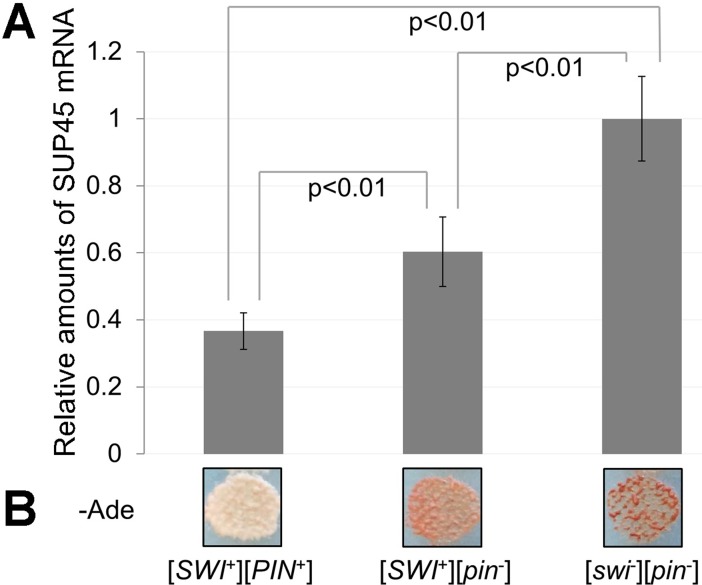
[*SWI*^+^] and [*PIN*^+^] interaction causes nonsense suppression by decreasing *SUP45* expression. (A) Real-time PCR analysis of *SUP45* mRNA levels in the [*SWI*^+^][*PIN*^+^], [*SWI*^+^][*pin*^-^], and [*swi*^-^][*pin*^-^] strains. The results are presented as the 2^-ΔΔC(t)^ ± the standard deviation (for details, see Materials and Methods). Significance levels are indicated. (B) Images illustrating the differences in growth between [*SWI*^+^][*PIN*^+^], [*SWI*^+^][*pin*^-^], and [*swi*^-^][*pin*^-^] strains on–Ade medium with 150 μM CuSO_4_. Images were obtained after 5 days of incubation.

In a previous work, we demonstrated that levels of *SUP45* mRNA expression and Sup45 production were 2–3 times higher in the [*nsi*^-^] strain than in [*NSI*^+^] [28]. Moreover, nonsense suppression does not manifest, when the [*NSI*^+^] strain is transformed by the centromeric plasmid containing *SUP45* under the control of endogenous promoter [29–31]. Thus, even two-fold increase in the level of *SUP45* expression completely prevents the appearance of nonsense suppression in the [*NSI*^+^] strains. To determine whether decreased expression of *SUP45* and nonsense suppression in the [*NSI*^+^] strain depend on the [*SWI*^+^] and [*PIN*^+^] prions, we compared *SUP45* mRNA levels in the [*SWI*^+^][*PIN*^+^], [*SWI*^+^][*pin*^-^], and [*swi*^-^][*pin*^-^] strains. RNA extraction, cDNA synthesis, and real-time PCR were performed as described in “Materials and Methods”. The data obtained show that *SUP45* mRNA levels in the [*swi*^-^][*pin*^-^] strain were approximately 1.5 and 2.5 times higher (p<0.01) than in the [*SWI*^+^][*pin*^-^] and [*SWI*^+^][*PIN*^+^] strains, respectively (Fig 6A). We may conclude that Swi1 is a positive regulator of *SUP45*, whereas the prion inactivation of Swi1 decreases the *SUP45* expression level. Importantly, *SUP45* expression was higher (p<0.01) in the [*SWI*^+^][*pin*^-^] strain than in [*SWI*^+^][*PIN*^+^] (Fig 6A). These data show that [*PIN*^+^] not only influences prion aggregation of Swi1 (Fig 5B), but also [*PIN*^+^] enhances the effect of [*SWI*^+^] on *SUP45* expression (Fig 6A). These effects correlate with the differences in growth on–Ade medium between the [*swi*^-^][*pin*^-^], [*SWI*^+^][*pin*^-^], and [*SWI*^+^][*PIN*^+^] strains (Fig 6B). Thus, interaction between [*SWI*^+^] and [*PIN*^+^] causes decreased *SUP45* expression and leads to nonsense suppression.

## Discussion

Previously, we performed a set of unsuccessful attempts to identify the [*NSI*^+^] factor using genetic approaches [19,21]. Genetic screening for the detection of prion structural genes may be useless because the overexpression of prion-forming proteins does not always lead to prion induction [16], and deletion screens are always incomplete, as they cannot be applied to essential genes. Here, for the first time, we identified protein determinants of an unknown prion factor using a proteomic approach. Two similar methods, TAPI and PSIA, based on the universal feature of amyloids to form detergent-resistant aggregates, were recently developed [22,24]. Both methods have some limitations and include the step of protein extraction from an agarose or polyacrylamide gel. Using a new gel-free modification of this proteomic approach named PSIA-LC-MALDI, we have shown that [*NSI*^+^] cells, in contrast to [*nsi*^-^], contain SDS-resistant aggregates of Swi1, Rnq1 and Mit1 proteins (S1 Table, S4–S6 Figs). Note, using the original PSIA method, we detected only Rnq1 protein in the SDS-resistant fraction of the [*NSI*^+^] strain (Fig 1, S3 Fig) and did not identify Swi1 or Mit1. These data suggest that PSIA-LC-MALDI is a more powerful method for the identification of proteins forming amyloid-like aggregates.

Deletion of *MIT1* does not affect the manifestation or maintenance of [*NSI*^+^] (Fig 4B); thus, we can conclude that *MIT1* is not a structural gene for [*NSI*^+^]. At the same time, our SDD-AGE experiment demonstrated that a small portion of Mit1-YFP protein forms SDS-resistant aggregates in both [*NSI*^+^] and [*nsi*^-^] cells. It is probable that Mit1 was not identified in proteomic screening in SDS-resistant fraction of the [*nsi*^-^] strain, because this protein is expressed in yeast at an extremely low level [32,33], and only a small portion of Mit1 forms SDS-resistant aggregates that may be detected at the lower limit of sensitivity of our PSIA-LC-MALDI approach. Mit1 is a transcriptional regulator of pseudohyphal growth [25], whose sequence contains a region extremely rich in asparagine (http://www.yeastgenome.org/locus/S000000733/protein). Based on the data obtained, we propose that Mit1 forms amyloid-like aggregates at physiological conditions, though the possible functional roles of these aggregates are unclear and may represent a subject for further study.

We have shown that the chimeric proteins Rnq1-CFP and Swi1-YFP form SDS-resistant aggregates in the [*NSI*^+^] strain, but not in [*nsi*^-^] (Figs 2A and 3A). Thus, the [*NSI*^+^] cells, in contrast to [*nsi*^-^], contain Rnq1 and Swi1 proteins in their prion forms. Surprisingly, the elimination of the [*PIN*^+^] prion in [*NSI*^+^] cells causes a strong decrease in the nonsense suppression level (Fig 2B). The weak suppressor phenotype was stably inherited in the strain that lost the [*PIN*^+^] factor and efficiently eliminated by GuHCl. These data suggest that the strong suppressor phenotype in [*NSI*^+^] strains is determined not only by [*PIN*^+^] but also by another prion, [*SWI*^+^], which was identified by PSIA-LC-MALDI. Elimination of [*SWI*^+^] causes the complete loss of all manifestations of the [*NSI*^+^] phenotype (Fig 3B). Taking into consideration that the strong suppressor phenotype manifests only in [*PIN*^+^][*SWI*^+^] strains, we conclude that the heritable trait detected in our strains is the result of the interaction of [*PIN*^+^] and [*SWI*^+^] prions (Fig 5A). Our data showing that [*PIN*^+^] enhances Swi1-YFP aggregation (Fig 5B) strongly support this conclusion (Fig 5B). At first glance, the appearance of a heritable trait related to a prion-prion interaction is surprising, because previous studies have shown that coexisting prion polymers typically do not physically interact [16,34]. Moreover, it was recently shown that aggregates of Swi1-YFP and Rnq1-CFP do not colocalize in [*PIN*^+^][*SWI*^+^] cells [16]. Nevertheless, although [*PIN*^+^] and [*SWI*^+^] prions show no colocalization (Fig 5C), they exhibit a functional interaction that is mediated by other components of the proteomic network and can be monitored by the level of nonsense suppression.

Prion conversion may lead not only to protein inactivation but also to the acquisition of novel functions. For example, Rnq1 protein only in its prion state causes hyperphosphorylation of Pin4 [35]. The authors of this work suggest that [*PIN*^+^] prion could serve as an epigenetic switch to promote the post-translational modification of yeast proteins. We have shown that Rnq1 in the [*PIN*^+^] state increases [*SWI*^+^]-dependent nonsense suppression (Fig 5A). One cannot exclude the possibility that Rnq1 in its prion form causes posttranslational modification of Swi1 prion aggregates. On the other hand, it can be assumed that [*PIN*^+^] polymers may affect chaperone machinery which interacts with prion [*SWI*^+^] and can influence its properties. Our data according to that Sis1 chaperone is presented in fraction of SDS-resistant aggregates only in the [*PIN*^+^] [*SWI*^+^] strain (S1 Table) support this hypothesis.

**Fig 7:**
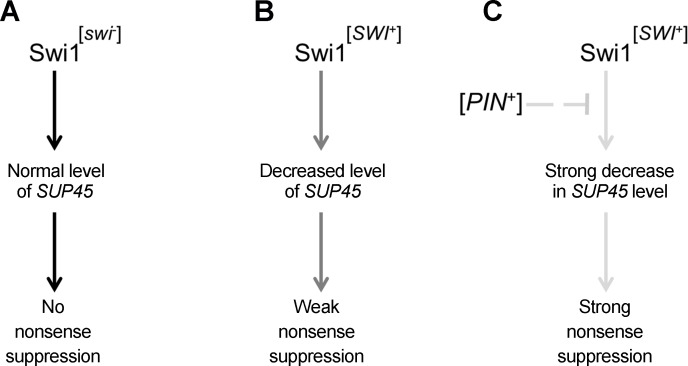
A scheme illustrating the influence of [*SWI*^+^] and [*PIN*^+^] interaction on nonsense suppression. (A) Swi1 normally acts as an activator of *SUP45* expression (black arrow). (B) Prion inactivation of Swi1 partially blocks activation of *SUP45* expression resulting in weak nonsense suppression (dark grey arrow). (C) [*PIN*^+^] indirectly enhances Swi1 inactivation in the [*SWI*^+^] strain (light grey dashed line), thereby blocking the Swi1-dependent activation of *SUP45* expression (light grey arrow).

Swi1 is a global transcriptional regulator that affects the transcription of a number of yeast genes [36,37]. We showed that Swi1 positively regulates the transcription of the *SUP45* gene (Fig 6A) that encodes the translation termination factor eRF1 [29]. Prion inactivation of Swi1 causes a decrease in *SUP45* expression that leads to the weak suppressor phenotype (Fig 6). [*PIN*^+^] increases [*SWI*^+^]-dependent inactivation of Sup45 and enhances nonsense suppression (Fig 5A and Fig 6). A scheme illustrating the effect of interaction between [*SWI*^+^] and [*PIN*^+^] on nonsense suppression is presented in Fig 7.

Overall, we can conclude that [*SWI*^+^] and [*PIN*^+^], like classical genes, demonstrate complementary interactions, and this prion-prion interaction causes heritable traits in *Saccharomyces cerevisiae*. [*SWI*^+^] is the key determinant of nonsense suppression, while [*PIN*^+^] does not cause nonsense suppression by itself, but strongly enhances the effect of [*SWI*^+^]. Thus, by analogy with monogenic and polygenic inheritance, in the framework of the prion concept we can distinguish “monoprionic” and “polyprionic” types of inheritance. We assume that new examples of polyprionic inheritance will be revealed using the proteomic methods for identification of prions.

## Materials and Methods

### Strains and growth conditions

Standard yeast genetic techniques, media, and cultivation conditions were used [38]. Yeast cultures were grown at 30°C. 150 μM copper sulfate (CuSO_4_) was added to synthetic and YPD media to induce the expression of genes under the *P*_*CUP1*_ promoter. To eliminate prions, yeast cultures were grown for three consecutive passages on the solid YPD medium in the presence of 5 mM Guanidine Hydrochloride (GuHCl). Growth on the selective medium containing 20 g/l galactose (Gal) as the sole carbon source was analyzed as described below. Yeast were grown for one day on the solid YPD medium and then passaged threefold, for one day per passage, on solid medium containing 20 g/l galactose as the sole carbon source at 30°C.

1-1-D931 [*NSI*^+^] and 1-1-1-D931 [*nsi*^-^] strains were described previously [18]. The genotype of these strains is *MAT*a *sup35*Δ::*HIS3 ade1-14 his3 leu2 lys2 ura3 trp1-289* [pU-Aβ-Sup35MC]. The 4-1-1-D931 [*NSI*^+^] and 1-4-1-1-D931 [*nsi*^-^] strains have the same genotype but contain the pL-Aβ-Sup35MC plasmid [18]. The pU-Aβ-Sup35MC and pL-Aβ-Sup35MC plasmids contain hybrid *Aβ-SUP35MC* gene under the control of the *P*_*CUP1*_ promoter, which compensates for *SUP35* deletion in the chromosome [18]. Presence of the [*NSI*^+^] factor was detected by suppression of the *ade1-14*_*UGA*_ mutation resulting in the growth of [*NSI*^+^] strains on the synthetic medium without adenine (–Ade). This medium was supplemented with 150 μM CuSO_4_ to overexpress *Aβ-SUP35MC* that is essential for the detection of differences in growth of [*NSI*^+^] and [*nsi*^-^] cells on–Ade medium.

The 5-1-1-D931 [*NSI*^+^] *rnq*Δ strain (*MAT*a *sup35*Δ::*HIS3 ade1-14 his3 leu2 lys2 ura3 trp1-289 rnq1*Δ::*KanMX* [pU-Aβ-Sup35MC]) contains a deletion of *RNQ1* chromosomal copy substituted with a *KanMX* cassette that provides resistance to the antibiotic geneticin (G418). This strain was obtained by the PCR-generated gene deletion technique [39], during which *KanMX* cassette flanked by 5’ and 3’ regions of *RNQ1* was PCR-amplified from the plasmid pFA6-kanMX4 with the primers FRNQ1deltaKanMX4 and RRNQ1deltaKanMX4 (first PCR), FRNQ1delta and RRNQ1delta (second PCR) (S2 Table) and transformed into the strain 1-1-D931 [*NSI*^+^]. Transformants were selected on YPD plates containing 200 mg/L G418 (Promega, USA). The deletion of *RNQ1* was PCR-verified with the primers FRNQ1deltach and RRNQ1deltach (S2 Table) in three independently obtained transformants. Analogously, the 11-1-1-D931 [*NSI*^+^] *swi1*Δ strain (*MAT*a *sup35*Δ::*HIS3 ade1-14 his3 leu2 lys2 ura3 trp1-289 swi1*Δ::*KanMX* [pU-Aβ-Sup35MC]) was obtained from 1-1-D931 [*NSI*^+^] strain. *KanMX* cassette flanking by sequences from promoter and middle region of *SWI1* was PCR-amplified from the plasmid pFA6-kanMX4 with the primers FSWI1deltaKanMX4 and RSWI1deltaKanMX4 (first PCR); and FSWI1delta and RSWI1delta (second PCR) (S2 Table). Deletion was verified by PCR with FSWI1deltach and RSWI1deltach (S2 Table) in three independently obtained transformants. The 2–936 [*NSI*^+^] *mit1*Δ strain (*MAT*a *sup35*Δ::*HIS3 ade1-14 his3 leu2 lys2 ura3 trp1-289 mit1*Δ::*KanMX* [pU-Aβ-Sup35MC]) was obtained by mating the *mit1*Δ strain (*MATα his3*Δ*1 leu2*Δ *lys2*Δ *ura3*) from the BY4742 deletion collection (Invitrogen, USA) to the 1-1-D931 [*NSI*^+^] strain followed with sporulation and dissection of the resulting diploids. *MIT1* deletion was PCR-verified with the primers FMIT1deltach and RMIT1deltach (S2 Table). The [*nsi*^-^] derivatives of the [*NSI*^+^] strains bearing deletions of the *MIT1*, *RNQ1* or *SWI1* genes were obtained by GuHCl treatment.

The D938 [*SWI*^+^][*PIN*^+^] diploid strain (*SUP35/sup35*Δ::*HIS3 ADE1/ade1-14 his*3*/his3 leu2/leu2 lys2/lys2 ura3/ura3 TRP1/trp1-289*) was obtained by mating the 1-1-D931 strain to BY4742 (*MATα his3*Δ*1 leu2*Δ *lys2*Δ *ura3*) (Invitrogen, USA) followed by elimination of the pU-Aβ-Sup35MC plasmid.

The 26-1-4-1-1-D931 [*swi*^-^][*PIN*^+^], 12-1-4-1-1-D931 [*SWI*^+^][*pin*^-^], and 16-1-4-1-1-D931 [*SWI*^+^][*PIN*^+^] strains were obtained by transformation of the 1-4-1-1-D931 [*swi*^-^][*pin*^-^] recipient yeast cells with 1-1-D931 [*SWI*^+^][*PIN*^+^] protein lysates (see “Transformation of yeast cells with protein lysates”).

### Plasmids

The pRNQ1-GFP (URA3) plasmid that contains chimeric *RNQ1-GFP* gene under the control of the copper-inducible P_*CUP1*_ promoter was described early [10]. The pCUP1-RNQ1-CFP(LEU2) plasmid contains *RNQ1* fused with the sequence encoding cyan fluorescent protein (CFP) under the control of the P_*CUP1*_. To obtain this plasmid, the fragment encoding CFP was PCR-amplified from the plasmid pDH5 (Yeast Resource Center, University of Washington, USA, http://depts.washington.edu/~yeastrc) with the primers FCFPSacII and RCFPSacI (S2 Table). Next, the sequence encoding green fluorescent protein (GFP) in the plasmid pRNQ1-GFP (URA3) [10] was substituted with PCR-amplified CFP digested with *Sac*I and *Sac*II. Finally, the *Xho*I-*Sac*I restriction fragment of the pRNQ1-CFP (URA3) plasmid containing P_*CUP1*_-RNQ1-CFP was inserted into the pRS415 multicopy vector [40].

pMIT1-MIT1-GFP(URA3) plasmid was constructed as follows. The Mit1-encoding sequence was PCR-amplified using the primers MIT1F and MIT1R (S2 Table) using YGPM21o12 plasmid of the YSC4613 yeast genomic library (Open Biosystems, USA) as a template. Next, PCR-amplified *MIT1* was inserted into the pRNQ1-GFP (URA3) plasmid by the *Bam*HI and *Sac*II digestion sites. As a result, pCUP-MIT1-GFP (URA3) was obtained. Further, *MIT1* promoter was PCR-amplified using MIT1_prom_F and MIT1_prom_R primers (S2 Table) and the YGPM21o12 plasmid as a template. The resulting P_*MIT1*_ sequence was inserted into the pCUP-MIT1-GFP (URA3) with digestion sites *Cla*I and *BamH*I.

The pCUP1-SWI1(1–297)-YFP(URA3) plasmid contains a Swi1(aa 1–297)-encoding sequence fused in-frame with the sequence encoding yellow fluorescent protein (YFP) under the control of P_*CUP1*_ promoter. To construct this plasmid, *SWI1* fragment was PCR-amplified using FSWI1(1)HindIII and RSWI1(889)BamHI primers (S2 Table) and the YGPM19p21 plasmid of YSC4613 (Open Biosystems, USA) as a template. Next, the PCR-amplified *SWI1* fragment was cloned into the pU-CUP1-YFP plasmid [41] with the digestion sites *Hind*III and *BamH*I. The p426GPD–SWI1YFP plasmid contains the sequence encoding full-length Swi1 fused in-frame with YFP under the control of a strong constitutive P_*GPD*_ promoter [16] was kindly provided by L.N. Mironova (St. Petersburg State University).

### Transformation of yeast cells with protein lysates

Transformation of yeast cells with total protein lyzate was performed as described previously [18]. To introduce [*PIN*^+^] or [*SWI*^+^] prions, the 1-4-1-1-D931 [*swi*^*-*^][*pin*^*-*^] spheroplasts were co-transformed with 1-1-D931 [*SWI*^+^][*PIN*^+^] protein lysate and pRNQ1-GFP (URA3) plasmid. The transformants were selected on–Leu–Ura medium with 1M sorbitol, tested of mating type and analyzed for presence of [*PIN*^+^] or [*SWI*^+^] prions as follows. To analyze the presence of [*PIN*^+^], the aggregation of Rnq1-GFP was analyzed by the fluorescence microscopy (see “Fluorescence microscopy”). The [*SWI*^+^] status of transformants was phenotypically detected by the growth on–Ade medium with 150 ΔM CuSO_4_. To confirm [*SWI*^+^] status of protein transformants the pRNQ1-GFP (URA3) plasmid was replaced with the pCUP1-SWI1(1–297)-YFP(URA3) plasmid, and fluorescence analysis of the Swi1(1–297)-YFP aggregation was performed.

### Proteomic screening and identification of amyloids (PSIA)

The PSIA (Proteomic Screening and Identification of Proteins) approach was described previously (for details, see [22]). In general, PSIA consists of three steps: (i) isolation of detergent-resistant protein aggregate fractions, (ii) separation of proteins from aggregates by two-dimensional difference gel electrophoresis (2D-DIGE), and (iii) identification of separated proteins. The isolation of proteins forming detergent-resistant aggregates is comprised of a series of ultracentrifugations of protein lysates at 151000 x g coupled to treatment with ionic detergents [22]. In this study, samples were treated with 1% sodium dodecyl sulfate (SDS). Additionally, 0.1% SDS was added to the sucrose cushion for ultracentrifugation followed the detergent treatment.

The proteins from the [*NSI*^+^] and [*nsi*^-^] strains were labeled at lysine residues with Cy5 and Cy3 fluorescent dyes, correspondingly, according to recommendations of the manufacturer. The proteins were dissolved in UTC buffer (8 M urea, 2 M thiourea, 4% CHAPS, and 30 mM TrisHCl pH 8.5) and separated by 2D-DIGE. Gel slices were washed twice with deionized water and washed once with 40% acetonitrile in 50 mM ammoniumbicarbonate solution. Next, dehydration was performed in 100% acetonitrile followed by removing of liquid and air-drying of gel slices. The dried samples were incubated for 4 h with 5 ml of sequencing grade trypsin (Promega) 5 mg/ml solution, 100 mM ammonium bicarbonate (pH 7.0) at 37°C. Peptides were extracted with 0,5 ml of 0.1% trifluoroacetic acid in water. Mass spectrometric peptide analysis was performed using an Ultraflextreme MALDI-TOF/TOF mass spectrometer (Bruker Daltonics, DE) equipped with an Nd laser (354 nm) in reflecto-mode (the mass range 700–4500 m/z). The matrix was α-cyano-4-hydroxycinnamic acid. Peak lists were generated by the flexAnalysis 3.2 software (Bruker Daltonics). Proteins were identified by Mascot software release version 2.4.2 (Matrix Science, http://www.matrixscience.com) in the database of National Center for Biotechnology Information (NCBI) [22].

### PSIA / Liquid chromatography coupled with mass-spectrometry (PSIA-LC-MALDI)

This variant of PSIA uses high-performance liquid chromatography coupled with mass-spectrometry. The first step, isolation of detergent-resistant protein aggregate fractions, was performed as described previously [22]. Isolated proteins were lyophilized using the vacuum concentrator Labconco CentriVap (Labconco, USA). Next, lyophilized samples were treated with formic acid (90%), dried in the vacuum concentrator Labconco CentriVap (Labconco, USA), solubilized in Tris-buffered saline (TBS), and boiled in SDS-PAGE loading buffer. Then, detergents and salts were removed from the samples using HiPPR Detergent Removal columns (Thermo Scientific, USA) and Zeba Desalting columns (Thermo Scientific, USA), respectively, according to the manufacturers’ protocols. Final samples (volume 50 μl, total protein concentration 0.2–0.4 mg/ml) were supplemented with 1 μl of freshly prepared 50 mM DTT in 50 mM ammonium bicarbonate, incubated for 15 min at 50°C, supplemented with 1 μl 100 mM iodoacetamide in 50 mM ammonium bicarbonate and incubated for 15 min at 20°C in the dark. Then the samples were supplemented with 1 μl DTT to inactivate iodoacetamide and 5 μl trypsin (10 ng/μl; Sigma) and incubated overnight at 37°C. The trypsin was inactivated by adding 0.5 μl 10% TFA followed by centrifuging for 30 min (20,000*g*, 4°C). The final peptide mixtures were loaded (1 μl) onto an Acclaim PepMap 300 HPLC reverse-phase column (150 mm, 75 μm, particle size 5 μm; Thermo Scientific, USA) and separated in an acetonitrile gradient (2–90%) during 45 min using an UltiMate 3000 UHPLC RSLC nano high-performance nanoflow liquid chromatograph (Dionex, USA). Peptide fractions were collected every 10 s and loaded onto a 384-sample MTP AnchorChip 800/384 microtiter plate (Bruker Daltonics) using spotter Proteineer fc II (Bruker Daltonics) [42].

Peptides were identified using the Ultraflextreme MALDI-TOF/TOF mass spectrometer (Bruker Daltonics, DE). MS-spectra for each peptide fraction were determined and analyzed using WARP-LC software. An array of unique peptides characterized by specific retention time, charge, and molecular weight was determined. MS/MS-analysis was performed for these peptides in fractions (spots) with maximal concentration (peak intensity) of these peptides. Match between the experimental spectra and corresponding proteins was analyzed using Mascot version 2.4.2 software (Matrix Science; http://www.matrixscience.com) in the UniProt database (http://www.uniprot.org/) restricted to *Saccharomyces cerevisiae*. As matrix, α-cyano-4-hydroxycinnamic acid was used. During analysis, preset parameters of “Mass tolerance” were used (precursor mass tolerance 100 ppm, fragment mass tolerance 0.9 Da). As a standard sample, Peptide Calibration Standard II 8222570 (Bruker Daltonics) was applied. Carboxymethylation of cysteine, partial oxidation of methionine, and one skipped trypsinolysis site were considered as permissible modifications [42]. The BioTools software (Bruker, Bremen, Germany) was used for manual validation of protein identification.

### Protein analysis

Preparation and fractionation of protein lysates by centrifugation were performed as described previously [43], with modifications. Total lysate was fractionated by centrifugation at 100 000 g for 20 min, 4°C. The supernatant was placed into a fresh tube, and the insoluble fraction was resuspended in an equal amount of lysis buffer. SDS, glycerol, β-mercaptoethanol, and Tris-HCl (pH 6.8) were added to each sample up to final concentrations of 3%, 10%, 3%, and 0.15 M, respectively. Resulting samples were heated at 95°C for 10 min and run on the standard SDS-polyacrylamide gel. Next, proteins were transferred onto Immobilon-P PVDF membrane (GE Healthcare, USA), reacted to antibodies against GFP [E385] (ab32146) (Abcam, Great Britain), and detected by Amersham ECL Prime Western Blotting Detection Reagent kit (GE Healthcare, USA).

Semi-Denaturing Detergent Agarose Gel Electrophoresis (SDD-AGE) [44,45] was performed with 1% agarose gel. Before separation, proteins were treated for 10 min with 1% SDS at room temperature. The separated proteins were transferred onto Immobilon-P PVDF membrane (GE Healthcare, USA). Proteins fused with CFP, GFP, and YFP were detected using monoclonal rabbit primary antibodies against GFP [E385] (ab32146) (Abcam, Great Britain) and the Amersham ECL Prime Western Blotting Detection Reagent kit (GE Healthcare, USA).

### Real-time PCR

RNA extraction was performed with “Trizol” reagent (“Invitrogene”, USA). Reverse transcription was carried out using SuperScript III cDNA synthesis kit (“Invitrogene”, USA). Real-time PCR was performed with primers (ACT1F, ACT1R, SUP45F, and SUP45R) and “TaqMan” probes (ACT1probe, SUP45probe) listed in S2 Table. The probes were conjugated with FAM (for *ACT1*) or R6G (for *SUP45*) fluorophores as well as with BHQ quencher (“Beagle”, Russian Federation). Actin-encoding gene *ACT1* was used as the reference gene. Results of real-time PCR were normalized with the 2^*-*ΔΔC(t)^ method [46]. This method uses ΔCt parameter indicating the difference in the intensities of signals between gene of interest (*SUP45*) and reference (*ACT1*). Next, ΔΔCt value, which is the difference between ΔCt parameters in experiment and control, is calculated. Finally, 2^*-*ΔΔC(t)^ is calculated. This value demonstrates the relative amounts of mRNAs of interest in comparison between the experiment and control samples. The results are presented as the means ± the standard deviations.

### Fluorescence microscopy

Fluorescence microscopy assays of GFP, YFP and CFP-fused proteins were performed with a Leica DM6000B microscope using GFP, YFP and CFP cubes and the Leica QWin Standart 3.2.0 software (Leica Microsystems GmBH, Germany).

## Supporting Information

S1 Fig Identification data of Ape1 protein from Fig 1. (PDF)

S2 Fig Identification data of Ape4 protein from Fig 1. (PDF)

S3 Fig Identification data of Rnq1 protein from Fig 1. (PDF)

S4 Fig Identification data of Rnq1 protein from S1 Table. (PDF)

S5 Fig Identification data of Swi1 protein from S1 Table. (PDF)

S6 Fig Identification data of Sis1 protein from S1 Table. (PDF)

S7 Fig Identification data of Mit1 protein from S1 Table. (PDF)

S8 Fig Fluorescence microscopy of Rnq1-GFP in the [*swi^-^*][*PIN^+^*] strain obtained by protein transformation and in the parental [*swi^-^*][*pin^-^*] strain. (TIF)

S9 Fig Fluorescence microscopy of Swi1(1–297)-YFP in the [*swi^-^*][*PIN^+^*], 12-1-4-1-1- [*SWI^+^*][*pin^-^*], and [*SWI^+^*][*PIN^+^*] strains obtained by protein transformation and in the parental [*swi^-^*][*pin^-^*] strain. (TIF)

S1 Table Proteins identified by PSIA-LC-MALDI in the 4-1-1-D931 [*NSI^+^*] and 1-4-1-1-D931 [*nsi^-^*] strains. (PDF)

S2 Table Oligonucleotides used in the study. (PDF)
