# Supplementary material for: Interaction of Prions Causes Heritable Traits in Saccharomyces cerevisiae
Source: PLoS Genet. 2016 Dec 27;12(12):e1006504. doi: 10.1371/journal.pgen.1006504 (PMC5189945; doi:10.1371/journal.pgen.1006504)
Supplement: S1 Fig — (PDF) [file pgen.1006504.s001.pdf]

Ape1

Sequence Name: Vacuolar aminopeptidase 1 OS=Saccharomyces cerevisiae (strain ATCC 204508 / S288c) GN=LAP4 PE=1 SV=2 AMPL_YEAST
MH+ (avg):1.008 MH+ (mono):1.008
Number of Peaks:70 Tolerance (Da):0.500

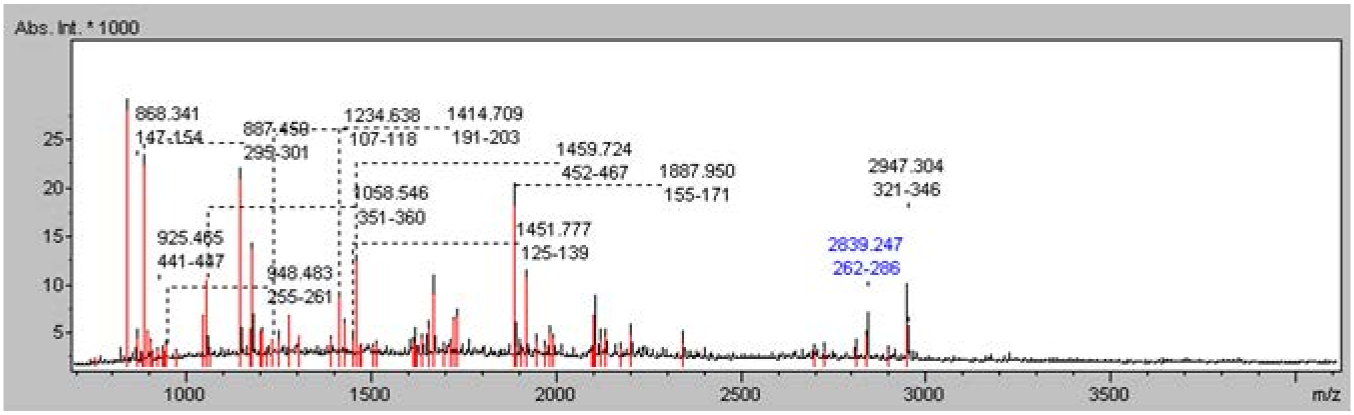

Sequence data:

Intensity Coverage: 25.1 % (102347 cnts)

Sequence Coverage MS: 31.7%
pI (isoelectric point): 5.5

10	20	30	40	50	60	70	80	90	100	110
MEEQREILEQ	LKKTLQMLTV	EPSKNNQIAN	EEKEKKENEN	SWCILEHNYE	DIAQEFIDFI	YKNPTTYHVV	SFFAELLDKH	NFKYLSEKSN	WQDSIGEDGG	KFYTIRNGTN
120	130	140	150	160	170	180	190	200	210	220
LSAFILGKNW	RAEKGVGVIG	SHVDALTVKL	KPVSFKDTAE	GYGRIAVAPY	GGTLNELWLD	RDLGIGGRLL	YKKKGTNEIK	SALVDSTPLP	VCRIPSLAPH	FGKPAEGPFD
230	240	250	260	270	280	290	300	310	320	330
KEDQTIPVIG	FPTPDEEGNE	PPTDDEKKSP	LFGKHCIHLL	RYVAKLAGVE	VSELIQMDLD	LFDVQKGTIG	GIGKHFLFAP	RLDDRLCSFA	AMIALICYAK	DVNTEESDLF
340	350	360	370	380	390	400	410	420	430	440
STVTLYDNEE	IGSLTRQGAK	GGLLESVVER	SSSAFTKKPV	DLHTVWANSI	ILSADVNHLY	NPNFPEVYLK	NHFPVPNVGI	TLSLDPNGHM	ATDVVGTALV	EELARRNGDK
450	460	470	480	490	500	510	520			
VQYFQIKNNS	RSGGTIGPSL	ASQTGARTID	LGIAQLSMHS	IRAATGSKDV	GLGVKFFNGF	FKHWRSVYDE	FGEL			

Display Parameter:

MH+ (mono): 1.008
MH+ (avg): 1.008
Tolerance (Da): 0.500
Number of Peaks: 70

Peaklist:

Peak	Mass	Intensity	Peak	Mass	Intensity	Peak	Mass	Intensity
1	756.361	2497.235	2	842.463	27836.221	3	868.341	4451.053
4	870.487	2944.384	5	881.418	3001.258	6	887.450	22121.741
7	899.489	5073.581	8	907.400	4096.799	9	910.429	3093.501
10	925.465	3246.481	11	937.456	3240.638	12	944.471	3236.240
13	948.483	3759.521	14	973.493	2955.394	15	1045.525	6617.985
16	1058.546	10504.588	17	1066.563	3338.229	18	1148.641	20696.677
19	1174.623	5224.606	20	1179.563	5804.317	21	1180.709	13654.950
22	1201.652	5197.123	23	1205.659	5159.545	24	1221.611	3464.555
25	1234.638	4391.179	26	1252.581	4319.937	27	1277.668	6655.790
28	1307.635	4743.387	29	1394.652	4390.287	30	1414.709	8748.020
31	1428.715	5956.262	32	1451.777	4225.589	33	1459.724	12315.149
34	1471.722	3647.968	35	1475.724	3494.654	36	1507.711	3612.272
37	1516.748	3941.582	38	1616.179	4145.445	39	1618.227	3900.777
40	1622.827	3564.359	41	1639.843	4415.299	42	1653.852	5467.353
43	1669.852	8924.778	44	1671.874	5617.426	45	1697.854	3611.247
46	1716.822	3909.739	47	1722.927	6312.676	48	1732.928	6699.442
49	1887.950	17930.920	50	1891.947	3624.966	51	1903.938	3881.268
52	1919.942	10585.954	53	1944.964	4002.110	54	1970.009	3564.453
55	1983.037	4910.585	56	1993.929	4373.747	57	2097.985	3283.860
58	2103.001	6651.549	59	2104.990	3718.755	60	2118.998	4182.502
61	2135.007	4557.258	62	2173.979	3460.054	63	2202.064	4786.190
64	2343.083	3811.258	65	2696.187	2966.077	66	2723.232	3054.878
67	2808.248	3268.594	68	2839.247	4681.644	69	2896.265	2778.179
70	2947.304	5971.590						
