# Supplementary material for: Interaction of Prions Causes Heritable Traits in Saccharomyces cerevisiae
Source: PLoS Genet. 2016 Dec 27;12(12):e1006504. doi: 10.1371/journal.pgen.1006504 (PMC5189945; doi:10.1371/journal.pgen.1006504)
Supplement: S2 Fig — (PDF) [file pgen.1006504.s002.pdf]

Ape4

Sequence Name:	Aspartyl aminopeptidase 4 OS=Saccharomyces cerevisiae (strain ATCC 204508 / S288c) GN=APE4 PE=1 SV=1 DNPEP_YEAST		
MH+ (avg):	1.008	MH+ (mono):	1.008
Number of Peaks:	47	Tolerance (Da):	0.500

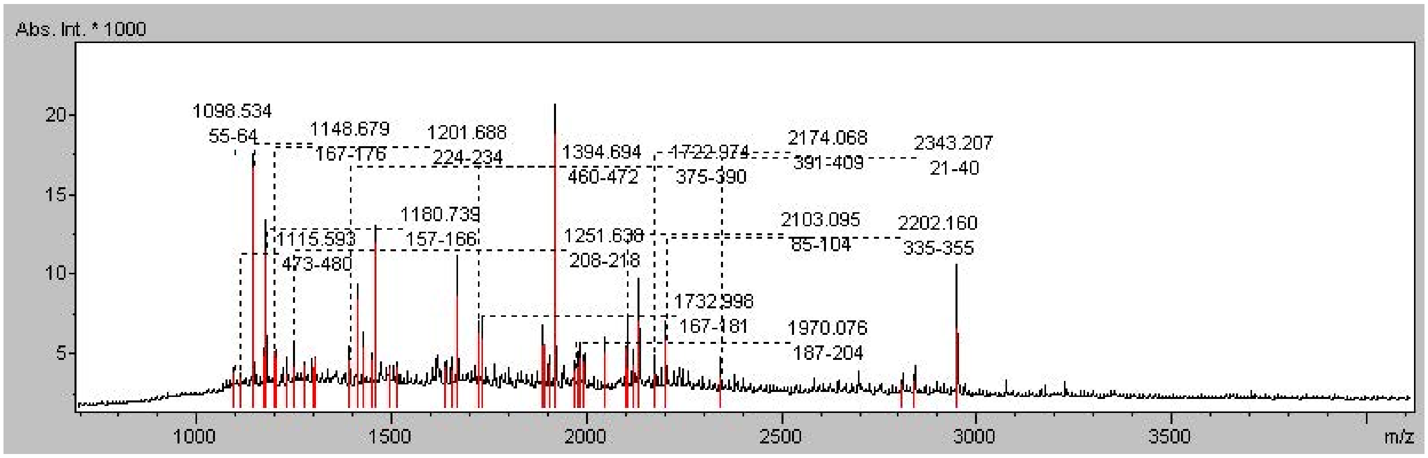

Sequence data:

Intensity Coverage:	31.6 % (84173 cnts)	Sequence Coverage MS:	39.2%
		pI (isoelectric point):	6.6

10	20	30	40	50	60	70	80	90	100	110
MFRIQLRTMS	SKTCKSDYPK	EFVSFLNSSH	SPYHTVHNIK	KHLVSNGFKE	LSERDSWAGH	VAQKGKYFVT	RNGSSIIAFA	VGGKWEPGNP	IAITGAHTDS	PALRIKPISK
120	130	140	150	160	170	180	190	200	210	220
RVSEKYLQVG	VETYGGAIWH	SWFDKDLGVA	GRVFVKDAKT	GKSIARLVDL	NRPLLKIPTL	AIHLDRDVNQ	KFEFNRETQL	LPIGGLQEDK	TEAKTEKEIN	NGEFTSIKTI
230	240	250	260	270	280	290	300	310	320	330
VQRHHAELLG	LIAKELAIDT	IEDIEDFELI	LYDHNASTLG	GFNDEFVFSG	RLDNLTSCFT	SMHGLTLAAD	TEIDRESGIR	LMACFDHEEI	GSSSAQGADS	NFLPNILERL
340	350	360	370	380	390	400	410	420	430	440
SILKGDGSDQ	TKPLFHSAIL	ETSAKSFFLS	SDVAHAVHPN	YANKYESQHK	PLLGGGPVIK	INANQRYMTN	SPGLVLVKRL	AEAAKVPLQL	FVVANDSPCG	STIGPILASK
450	460	470	480	490	500					
TGIRTLDLGN	PVLSMHSIRE	TGGSADLEFQ	IKLFKEFFER	YTSIESEIVV						

Display Parameter:

Sequence Name:	Aspartyl aminopeptidase 4 OS=Saccharomyces cerevisiae (strain ATCC 204508 / S288c) GN=APE4 PE=1 SV=1 DNPEP_YEAST		
MH+ (mono):	1.008	MH+ (avg):	1.008
Tolerance (Da):	0.500	Number of Peaks:	47

Peaklist:

Peak	Mass	Intensity	Peak	Mass	Intensity	Peak	Mass	Intensity
1	1098.534	3898.381	2	1115.593	3535.283	3	1148.679	16578.730
4	1174.653	4851.818	5	1179.587	5394.658	6	1180.739	11354.255
7	1201.688	5185.014	8	1205.690	4837.137	9	1235.621	3784.635
10	1251.638	4065.788	11	1252.620	4359.672	12	1277.707	4295.441
13	1299.633	4242.460	14	1307.682	4801.136	15	1394.694	4652.489
16	1414.751	8360.346	17	1428.764	5495.884	18	1451.833	4680.712
19	1459.769	11905.383	20	1496.780	4128.086	21	1516.785	4192.999
22	1639.918	4231.008	23	1654.909	4044.262	24	1669.910	8647.823
25	1671.916	4573.138	26	1722.974	6272.862	27	1732.998	5947.811
28	1888.035	5595.380	29	1892.024	5645.499	30	1904.023	4296.597
31	1920.031	18591.713	32	1970.076	4005.436	33	1977.042	4187.863
34	1983.125	4734.608	35	1994.031	4605.347	36	2045.943	5104.331
37	2103.095	5425.808	38	2105.093	4077.560	39	2118.093	4336.180
40	2133.045	3965.608	41	2135.109	7062.635	42	2174.068	3841.986
43	2202.160	5845.170	44	2343.207	3564.056	45	2807.434	3170.576
46	2839.414	3375.718	47	2947.488	6642.416			
