# Supplementary material for: Interaction of Prions Causes Heritable Traits in Saccharomyces cerevisiae
Source: PLoS Genet. 2016 Dec 27;12(12):e1006504. doi: 10.1371/journal.pgen.1006504 (PMC5189945; doi:10.1371/journal.pgen.1006504)
Supplement: S3 Fig — (PDF) [file pgen.1006504.s003.pdf]

Rnq1

Sequence Name:	[PIN+] prion protein RNQ1 OS=Saccharomyces cerevisiae (strain ATCC 204508 / S288c) GN=RNQ1 PE=1 SV=2 RNQ1_YEAST		
MH+ (avg):	1.008	MH+ (mono):	1.008
Number of Peaks:	70	Tolerance (Da):	0.500

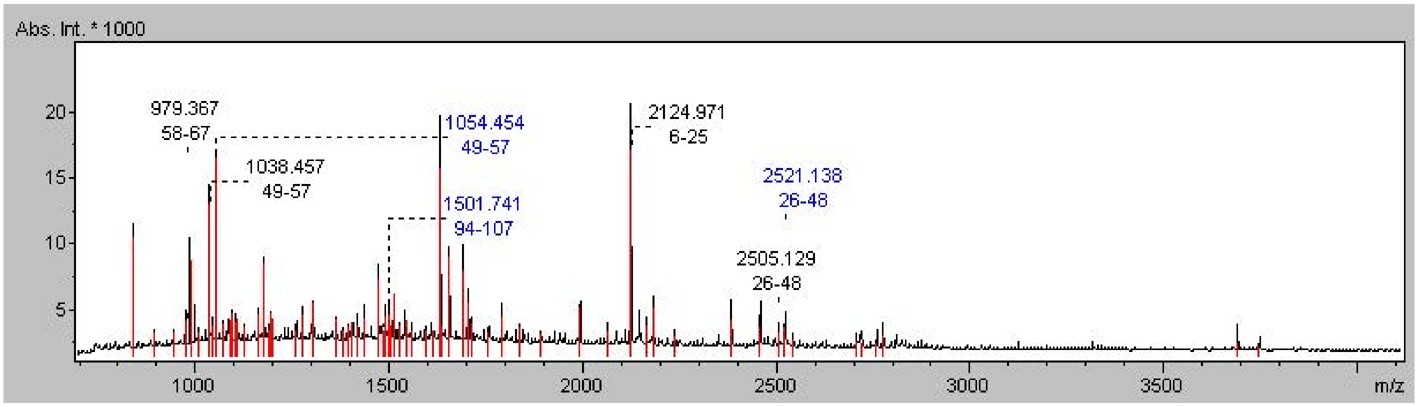

Sequence data:

Intensity Coverage:	18.0 % (61788 cnts)	Sequence Coverage MS:	18.8%
		pI (isoelectric point):	6.2

10	20	30	40	50	60	70	80	90	100	110
MDTDKLISEA	ESHFSQGNHA	EAVAKLTSAA	QSNPNDEQMS	TIESLIQKIA	GYVMDNRSGG	SDASQDRAAG	GGSSFMNTLM	ADSKGSSQTQ	LGKLALLATV	MTHSSNKGSS
**120**	**130**	**140**	**150**	**160**	**170**	**180**	**190**	**200**	**210**	**220**
NRGFDVGTVM	SMLSGSGGGS	QSMGASGLAA	LASQFFKSGN	NSQGQGQGQG	QGQGQGQGQG	QGSFTALASL	ASSFMNSNNN	NQQGQNQSSG	GSSFGALASM	ASSFMHSNNN
**230**	**240**	**250**	**260**	**270**	**280**	**290**	**300**	**310**	**320**	**330**
QNSNNSQQGY	NQSYQNGNQN	SQGYNNQQYQ	GGNGGYQQQQ	GQSGGAFSSL	ASMAQSYLGG	GQTQSNQQQY	NQQGQNNQQQ	YQQQGQNYQH	QQQGQQQQQG	HSSSFSALAS
**340**	**350**	**360**	**370**	**380**	**390**	**400**	**410**			
MASSYLGNNS	NSNSSYGGQQ	QANEYGRPQQ	NGQQQSNEYG	RPQYGGNQNS	NGQHESFNFS	GNFSQQNNNG	NQNRY			

Display Parameter:

MH+ (mono):	1.008	MH+ (avg):	1.008
Tolerance (Da):	0.500	Number of Peaks:	70

Peaklist:

Peak	Mass	Intensity	Peak	Mass	Intensity	Peak	Mass	Intensity
1	842.458	10410.735	2	899.476	3208.702	3	945.496	3223.352
4	979.367	4220.518	5	990.461	8696.353	6	1010.475	3210.276
7	1036.481	6668.434	8	1038.457	12931.359	9	1045.514	3722.654
10	1054.454	16250.402	11	1074.481	3908.786	12	1090.480	4190.823
13	1095.482	4635.366	14	1107.493	4064.462	15	1111.476	4286.230
16	1130.507	3610.781	17	1165.533	4614.206	18	1179.554	8404.953
19	1192.549	3390.920	20	1198.658	4682.768	21	1201.614	4233.699
22	1259.630	3427.699	23	1277.658	4621.105	24	1307.625	5480.034
25	1365.595	4250.796	26	1384.642	3414.541	27	1398.705	3580.625
28	1407.661	3999.457	29	1421.585	3826.448	30	1437.756	4243.308
31	1475.707	7222.369	32	1488.675	3399.802	33	1491.691	3476.054
34	1493.685	4456.171	35	1501.741	4538.552	36	1507.699	3775.114
37	1515.692	6145.364	38	1528.704	3759.210	39	1544.633	4218.455
40	1558.779	3411.509	41	1596.740	3302.838	42	1612.710	3400.988
43	1634.667	15536.328	44	1638.779	3793.876	45	1657.741	8958.275
46	1691.687	7918.852	47	1707.721	5480.960	48	1716.805	3847.186
49	1757.842	3224.439	50	1790.836	4428.648	51	1791.657	3245.298
52	1838.877	3616.689	53	1890.911	3151.412	54	1993.910	5120.962
55	2064.079	3304.195	56	2124.971	17019.357	57	2162.924	3773.266
58	2181.991	5135.544	59	2239.023	2961.307	60	2383.878	4186.797
61	2457.155	3594.128	62	2505.129	3258.907	63	2521.138	3568.555
64	2543.107	2688.567	65	2705.058	2574.039	66	2717.020	2447.325
67	2757.157	2443.961	68	2773.163	2966.561	69	3686.240	2439.016
70	3743.238	2195.832						
