# Supplementary material for: Interaction of Prions Causes Heritable Traits in Saccharomyces cerevisiae
Source: PLoS Genet. 2016 Dec 27;12(12):e1006504. doi: 10.1371/journal.pgen.1006504 (PMC5189945; doi:10.1371/journal.pgen.1006504)
Supplement: S4 Fig — (PDF) [file pgen.1006504.s004.pdf]

Rnq1

Sequence Name:	[PIN+] prion protein RNQ1 OS=Saccharomyces cerevisiae (strain ATCC 204508 / S288c) GN=RNQ1 PE=1 SV=2 RNQ1_YEAST
MH+ (mono):	1.008
MH+ (avg):	1.008
Tolerance (Da):	0.900
Number of Peaks:	1266

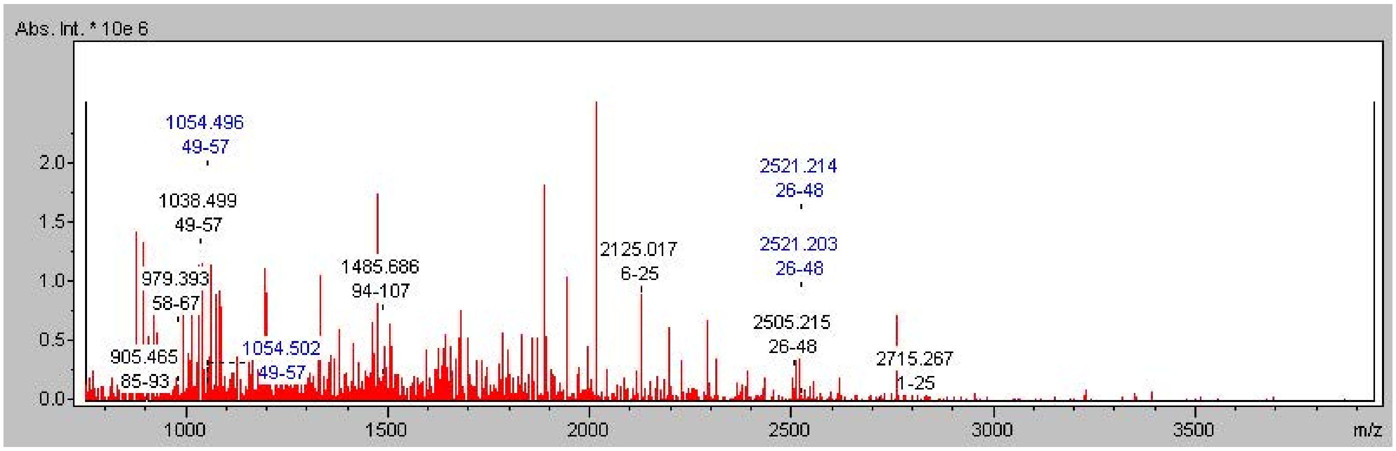

Sequence data:

Intensity Coverage:	2.3 % (3218274 cnts)	Sequence Coverage MS:	22.2%
Sequence Coverage MS/MS:	22.2%	pI (isoelectric point):	6.2

10	20	30	40	50	60	70	80	90	100
MDTDKLISEA	ESHFSQGNHA	EAVAKLTSAA	QSNPNDEQMS	TIESLIQKIA	GYVMDNRSGG	SDASQDRAAG	GGSSFMNTLM	ADSKGSSQTQ	LGKLALLATV

110	120	130	140	150	160	170	180	190	200
MTHSSNKGSS	NRGFDVGTVM	SMLSGSGGGS	QSMGASGLAA	LASQFFKSGN	NSQGQGQGQG	QGQGQGQGQG	QGSFTALASL	ASSFMNSNNN	NQQGQNQSSG

210	220	230	240	250	260	270	280	290	300
GSSFGALASM	ASSFMHSNNN	QNSNNSQQGY	NQSYQNGNQN	SQGYNNQQYQ	GGNGGYQQQQ	GQSGGAFSSL	ASMAQSYLGG	GQTQSNQQQY	NQQGQNNQQQ

310	320	330	340	350	360	370	380	390	400
YQQQGQNYQH	QQQGQQQQQG	HSSSFSALAS	MASSYLGNNS	NSNSSYGGQQ	QANEYGRPQQ	NGQQQSNEYG	RPQYGGNQNS	NGQHESFNFS	GNFSQQNNNG

410
NQNRY
