# Supplementary material for: Interaction of Prions Causes Heritable Traits in Saccharomyces cerevisiae
Source: PLoS Genet. 2016 Dec 27;12(12):e1006504. doi: 10.1371/journal.pgen.1006504 (PMC5189945; doi:10.1371/journal.pgen.1006504)
Supplement: S5 Fig — (PDF) [file pgen.1006504.s005.pdf]

Swi1

Sequence Name:	SWI/SNF chromatin-remodeling complex subunit SWI1 OS=Saccharomyces cerevisiae (strain ATCC 204508 / S288c) GN=SWI1 PE=1 SV=1 SWI1_YEAST		
		MH+ (mono):	1.008
MH+ (avg):	1.008	Tolerance (Da):	0.900
Number of Peaks:	1266		

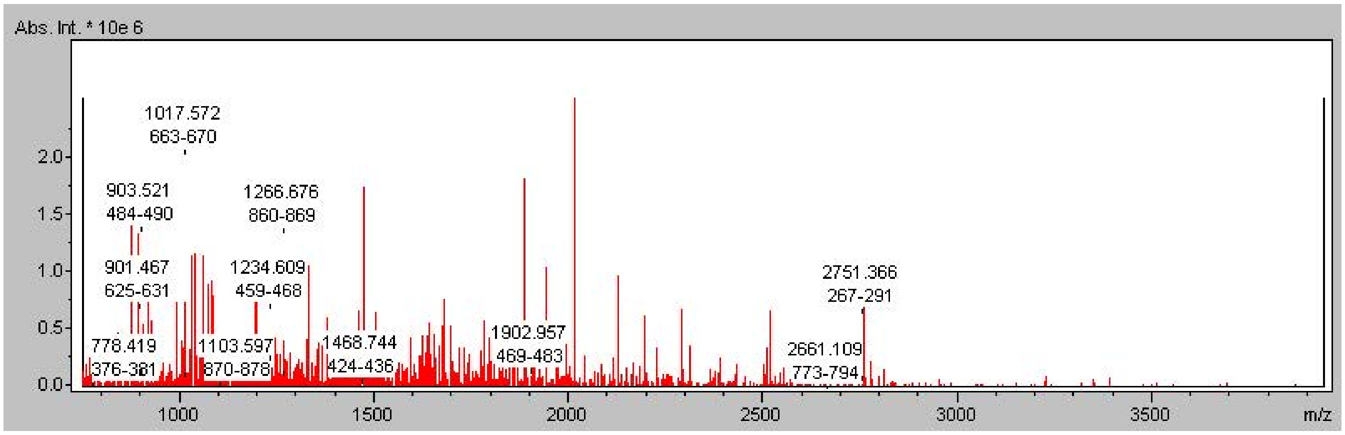

Sequence data:

Intensity Coverage:	0.6 % (843590 cnts)	Sequence Coverage MS:	10.0%
Sequence Coverage MS/MS:	10.0%	pI (isoelectric point):	9.4

10	20	30	40	50	60	70	80	90	100
MDFFNLNNNN	NNNNTTTTTT	TTNNNNTNNN	NTNNNNNPAN	NTNNNNSTGH	SSNTNNNTNN	NNTNTGASGV	DDFQNFFDPK	PFDQNLDSNN	NNSNSNNNDN
110	120	130	140	150	160	170	180	190	200
NNSNTVASST	NFTSPTAVVN	NAAPANVTGG	KAANFIQNQS	PQFNSPYDSN	NSNTNLNSLS	PQAILAKNSI	IDSSNLPLQA	QQQLYGGNNN	NNSTGIANDN
210	220	230	240	250	260	270	280	290	300
VITPHFITNV	QSISQNSSSS	TPNTNSNSTP	NANQQFLPFN	NSASNNGNLT	SNQLISNYAA	SNSMDRSSSA	SNEFVPNTSD	NNNNSNNHNM	RNNSNNKTSN
310	320	330	340	350	360	370	380	390	400
NNNVTAVPAA	TPANTNNSTS	NANTVFSERA	AMFAALQQKQ	QQRFQALQQQ	QQQQQNQQQQ	NQQPQQQQQQ	QQNPKFLQSQ	RQQQQRSILQ	SLNPALQEKI
410	420	430	440	450	460	470	480	490	500
STELNNKQYE	LFMKSLIENC	KKRNMPLQSI	PEIGNRKINL	FYLYMLVQKF	GGADQVTRTQ	QWSMVAQRLQ	ISDYQQLESI	YFRILLPYER	HMISQEGIKE
510	520	530	540	550	560	570	580	590	600
TQAKRIFLQQ	FLQELLKKVQ	QQQQAAALAN	ANNNINSASS	APTPAAPGAS	VPATAAPGTE	AGIVPVSANT	PKSLNSNINI	NVNNNNIGQQ	QVKKPRKQRV
610	620	630	640	650	660	670	680	690	700
KKKTKKELEL	ERKEREDFQK	RQQKLLEDQQ	RQQKLLLETK	LRQQYEIELK	KLPKVYKRSI	VRNYKPLINR	LKHYNGYDIN	YISKIGEKID	SNKPIFLFAP
710	720	730	740	750	760	770	780	790	800
ELGAINLHAL	SMSLQSKNLG	EINTALNTLL	VTSADSNLKI	SLVKYPELLD	SLAILGMNLL	SNLSQNVVPY	HRNTSDYYYE	DAGSNQYYVT	QHDKMVDKIF
810	820	830	840	850	860	870	880	890	900
EKVNNNATLT	PNDSNDEKVT	ILVDSLTGNQ	LPTPTPTEME	PDLDTECFIS	MQSTSPAVKQ	WDLLPEPIRF	LPNQFPLKIH	RTPYLTSLKK	IKDEIDDPFT
910	920	930	940	950	960	970	980	990	1000
KINTRGAEDP	KVLINDQLST	ISMILRNISF	SDNNSRIMSR	NFYLKRFISD	LLWLVLIHPE	NFTCNRKILN	FKKDLVIVLS	NISHLLEIAS	SIDCLLILIL
1010	1020	1030	1040	1050	1060	1070	1080	1090	1100
VISFGQPKLN	PMASSSSFGS	ESLTFNEFQL	QWGKYQTFGV	DILAKLFSLE	KPNLNYFKSI	LLNKNTGNNL	YDRNSNNNHK	DKKLLRRLLN	LYNDNNKNNN
1110	1120	1130	1140	1150	1160	1170	1180	1190	1200
NRHNLLNDVV	SFLFSAIPLQ	QVLSQSADPS	LLIDQFSPVI	SQSLTSILVI	VQKILPLSNE	VFEISENNSD	SNSNNNGNKD	SSFNFNKNLP	FVWLSSEENI
1210	1220	1230	1240	1250	1260	1270	1280	1290	1300
GSGLLKLSEI	ILNINNSTSK	NTLLQQQNYS	KVLLPSINIS	CVQLIKCLVE	KSICFENCLN	NDPEILKKIA	SIPNLFPTDL	EIFQLFTNPS	VDIQIINQYQ
1310	1320								
LLYNLKNDIL	TNLE								
