# Supplementary material for: Interaction of Prions Causes Heritable Traits in Saccharomyces cerevisiae
Source: PLoS Genet. 2016 Dec 27;12(12):e1006504. doi: 10.1371/journal.pgen.1006504 (PMC5189945; doi:10.1371/journal.pgen.1006504)
Supplement: S6 Fig — (PDF) [file pgen.1006504.s006.pdf]

Sis1

Sequence Name:	Protein SIS1 OS=Saccharomyces cerevisiae (strain ATCC 204508 / S288c) GN=SIS1 PE=1 SV=1 SIS1_YEAST		
		MH+ (mono):	1.008
MH+ (avg):	1.008	Tolerance (Da):	0.900
Number of Peaks:	1266		

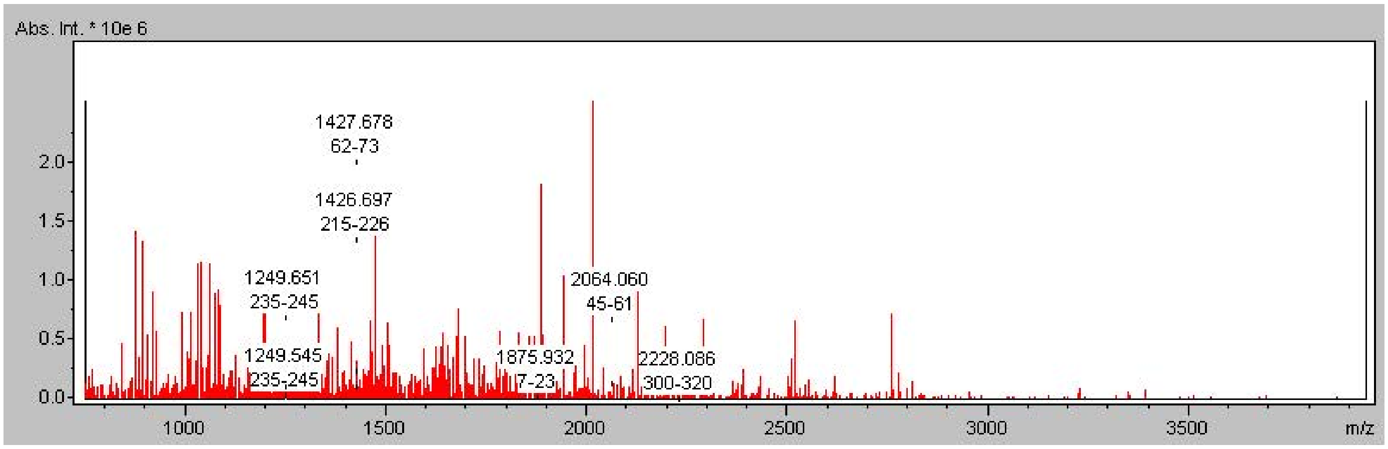

Sequence data:

Intensity Coverage:	0.3 % (472052 cnts)	Sequence Coverage MS:	25.6%
Sequence Coverage MS/MS:	25.6%	pI (isoelectric point):	9.4

10	20	30	40	50	60	70	80	90	100
MVKETKLYDL	LGVSPSANEQ	ELKKGYRKAA	LKYHPDKPTG	DTEKFKEISE	AFEILNDPQK	REIYDQYGLE	AARSGGPSFG	PGGPGGAGGA	GGFPGGAGGF
**110**	**120**	**130**	**140**	**150**	**160**	**170**	**180**	**190**	**200**
SGGHAFSNED	AFNIFSQFFG	GSSPFGGADD	SGFSFSSYPS	GGGAGMGGMP	GGMGGMHGGM	GGMPGGFRSA	SSSPTYPEEE	TVQVNLPVSL	EDLFVGKKKS
**210**	**220**	**230**	**240**	**250**	**260**	**270**	**280**	**290**	**300**
FKIGRKGPHG	ASEKTQIDIQ	LKPGWKAGTK	ITYKNQGDYN	PQTGRRKTLQ	FVIQEKSHPN	FKRDGDDLIY	TLPLSFKESL	LGFSKTIQTI	DGRTLPLSRV
**310**	**320**	**330**	**340**	**350**	**360**				
QPVQPSQTST	YPGQGMPTPK	NPSQRGNLIV	KYKVDYPISL	NDAQKRAIDE	NF				
