# Supplementary material for: Interaction of Prions Causes Heritable Traits in Saccharomyces cerevisiae
Source: PLoS Genet. 2016 Dec 27;12(12):e1006504. doi: 10.1371/journal.pgen.1006504 (PMC5189945; doi:10.1371/journal.pgen.1006504)
Supplement: S7 Fig — (PDF) [file pgen.1006504.s007.pdf]

Mit1

Sequence Name: Transcriptional regulator MIT1 OS=Saccharomyces cerevisiae (strain ATCC 204508 / S288c) GN=MIT1 PE=1 SV=1 MIT1_YEAST

MH+ (mono): 1.008

MH+ (avg): 1.008

Tolerance (Da): 0.900

Number of Peaks: 1266

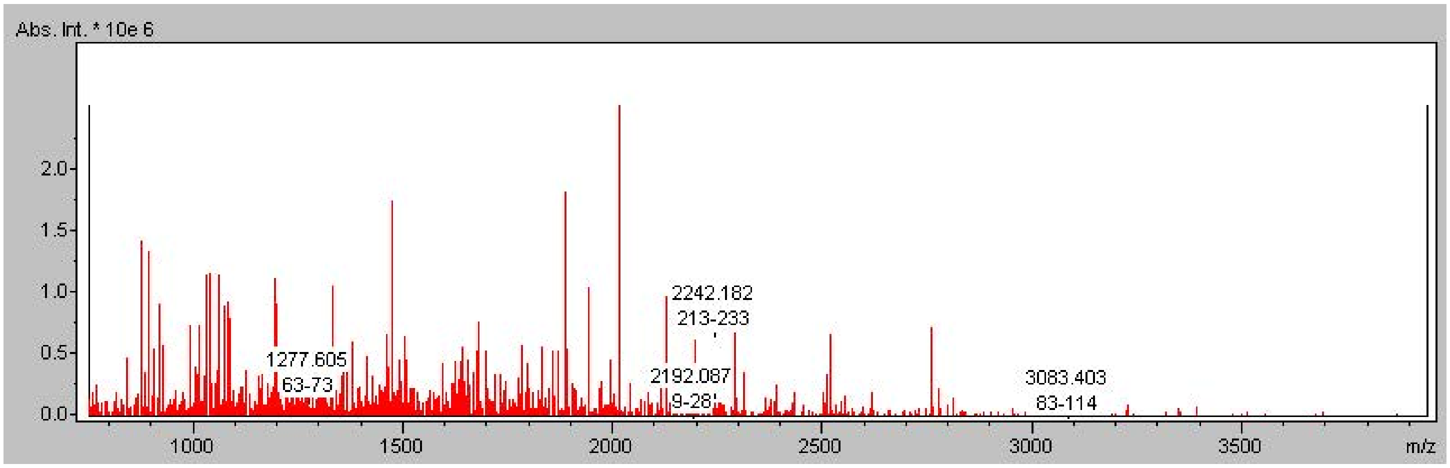

Sequence data:

Intensity Coverage: 0.2 % (281329 cnts)

Sequence Coverage MS: 12.6%

Sequence Coverage MS/MS: 12.6%

pI (isoelectric point): 6.7

10	20	30	40	50	60	70	80	90	100	110	120
MDIEPTFKGY	IEDEDDALLI	LQATLDGKLK	HIPRRPYEIE	RPYLIVSGSI	FVFIEEISGI	KRWTDGVSWS	PSRISGKFLI	YKELDKENAG	SNANATSSGS	TDSAVITDGT	SGARNNPSSS
**130**	**140**	**150**	**160**	**170**	**180**	**190**	**200**	**210**	**220**	**230**	**240**
KIKLPPLKNH	QFDLPPTMGH	SSFESEQDTS	ISPSNRSNLP	LKYTGLVKKT	ISVKLKRPPF	NSIENLHIVS	YYSVKDIKQN	CLVTPKASPF	LKDVRPSQEL	IVAMGNTTLG	NVKNNSTTTG
**250**	**260**	**270**	**280**	**290**	**300**	**310**	**320**	**330**	**340**	**350**	**360**
NGPNNINNKS	NSSTPLNTVI	STNNNSANIN	AAGSNQFTSA	NKNYYYKNDE	SSGYPITQFA	PALPSTTLMY	TANPPYITQS	PDNTNATGMN	THVNNNNNNS	NNSSNSNNSN	NNNNNNNNNN
**370**	**380**	**390**	**400**	**410**	**420**	**430**	**440**	**450**	**460**	**470**	**480**
NNNNNNINNI	NNVNTNAGNG	NNPNRFHNAS	FAYNTTGDFI	NPQQQGQISY	PFYYTTIPIN	NPNYYTTQPP	NPVTNASTNE	NQGYSTSSTQ	HPYYGHPTES	QSASAAAGAT	GTPGTAENVL
**490**	**500**	**510**	**520**	**530**	**540**	**550**	**560**	**570**	**580**	**590**	**600**
PVSSMQPLLH	QANNNSASSA	TSTAPYPVYS	MNVNVPYYNS	SASAYKRAQE	NTTSNTNAEP	SGATSTNSGT	MLSNPAYANS	QYTPSQVYYQ	GFPQYAMASA	QNPSMYQHQH	QHPLPTVYPI
**610**	**620**	**630**	**640**	**650**	**660**	**670**					
ATPQQNIMSS	GHTLSTIGSD	PQHHHYQQEP	NDHKNFAMGH	ANNNILNITN	NDTMNNLNTN	TSTTTQ					
