# Supplementary figures and images for: Interaction of Prions Causes Heritable Traits in Saccharomyces cerevisiae
Source: PLoS Genet. 2016 Dec 27;12(12):e1006504. doi: 10.1371/journal.pgen.1006504 (PMC5189945; doi:10.1371/journal.pgen.1006504)

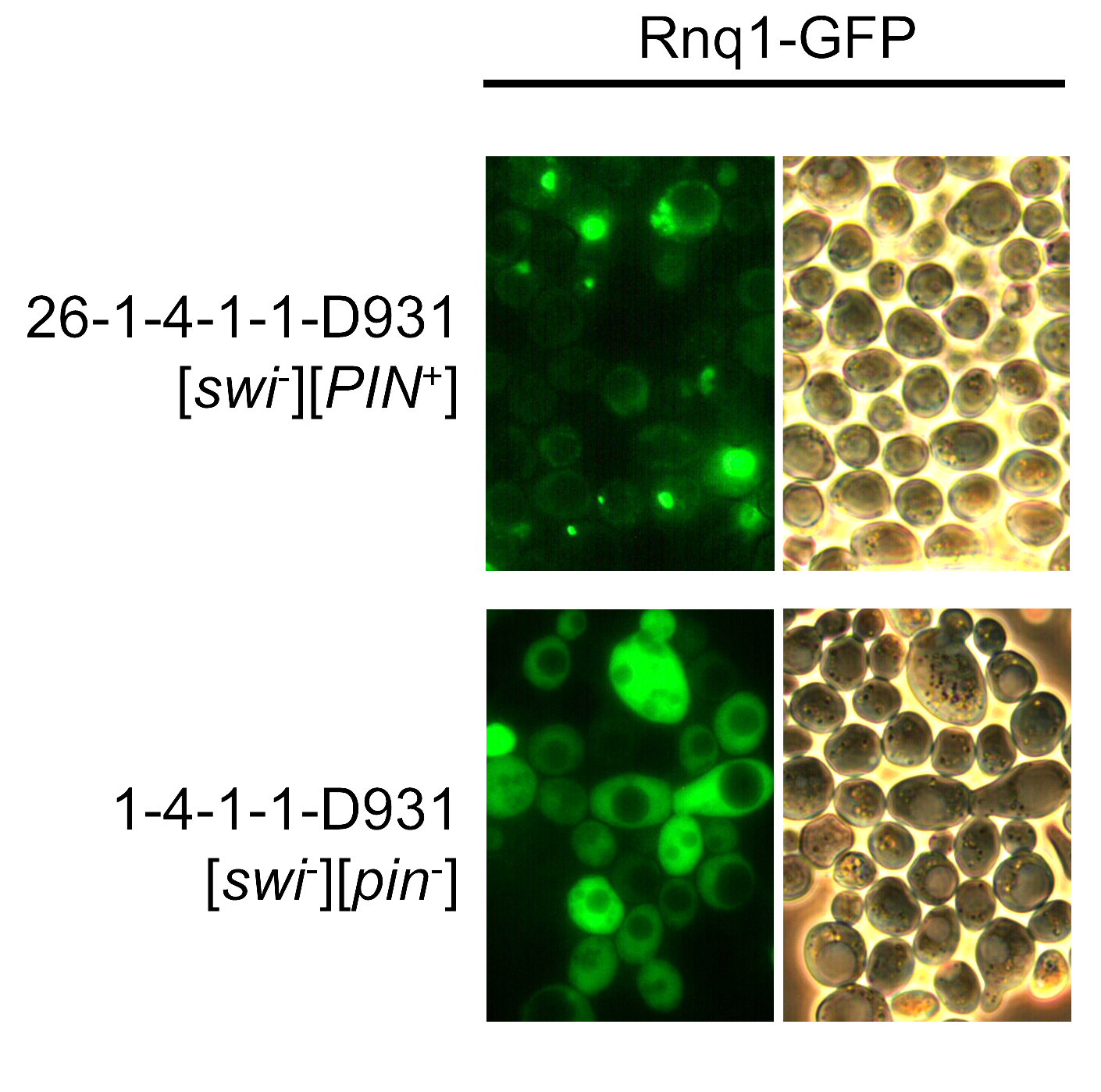

Supplement: S8 Fig — (TIF) [file pgen.1006504.s008.tif]

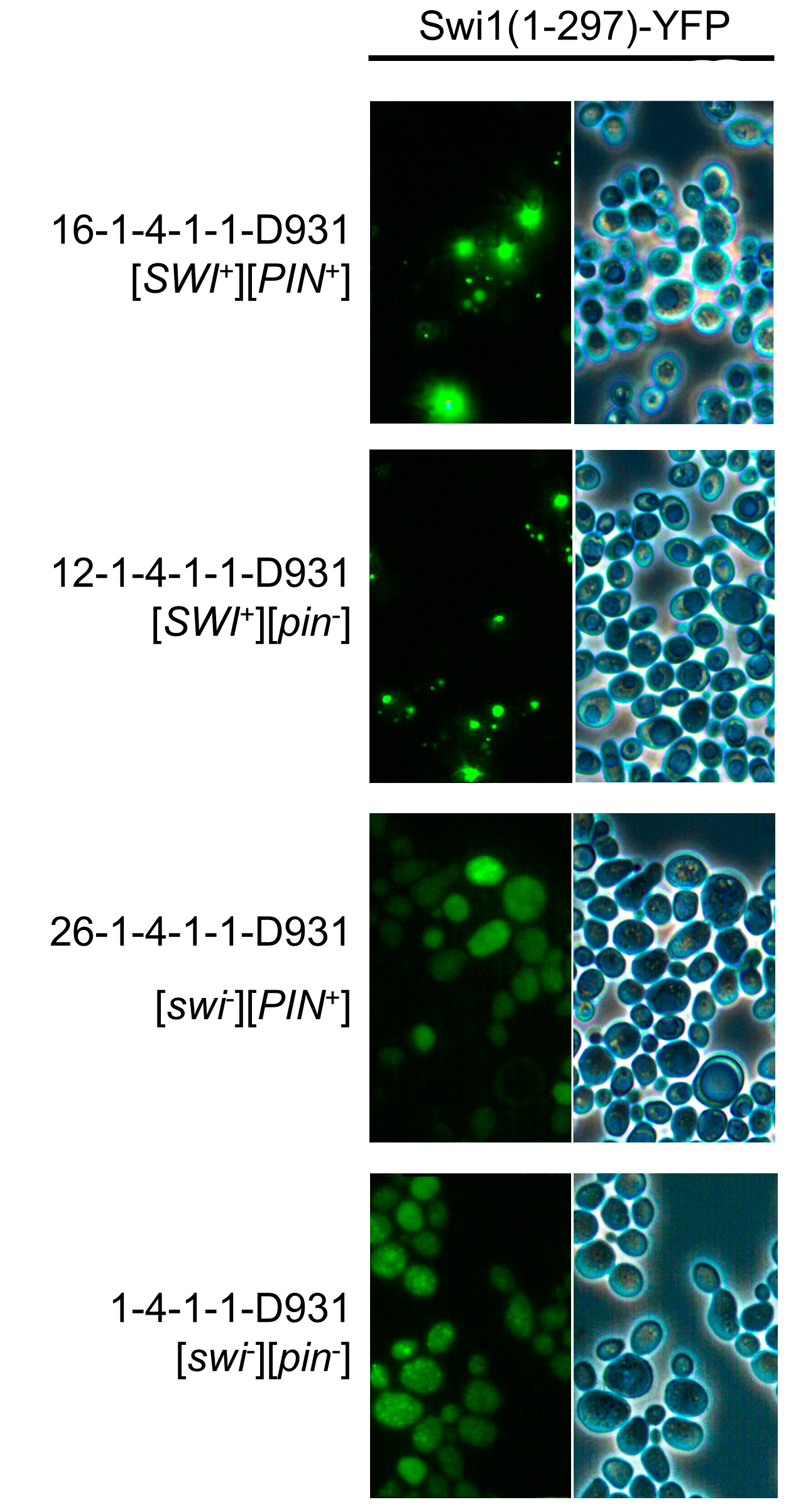

Supplement: S9 Fig — (TIF) [file pgen.1006504.s009.tif]
