# Supplementary material for: Interaction of Prions Causes Heritable Traits in Saccharomyces cerevisiae
Source: PLoS Genet. 2016 Dec 27;12(12):e1006504. doi: 10.1371/journal.pgen.1006504 (PMC5189945; doi:10.1371/journal.pgen.1006504)
Supplement: S1 Table — (PDF) [file pgen.1006504.s010.pdf]

S1 Table. Proteins identified by PSIA-LC-MALDI in the 4-1-1-D931 [NSI+] and 1-4-1-1-D931 [nsi-] strains.

**Protein**	**Score ([*NSI*$^+$])**	**Score ([*nsi*$^-$])**	**Function**
FAS2_YEAST	965	101	Fatty acid synthase subunit alpha
NSR1_YEAST	630	1592	Nuclear localization sequence-binding protein
AMPL_YEAST	553	1035	Vacuolar aminopeptidase 1
RIM1_YEAST	510	285	Single-stranded DNA-binding protein RIM1, mitochondrial
YRF11_YEAST	469	130	Y' element ATP-dependent helicase protein 1 copy 1
RNQ1_YEAST	460	not found	[PIN+] prion protein RNQ1
PYR1_YEAST	419	71	Protein URA2
GAS1_YEAST	391	778	1,3-beta-glucanosyltransferase GAS1
ECM33_YEAS2	349	470	Cell wall protein ECM33
PMA1_YEAST	348	276	Plasma membrane ATPase 1
BGL2_YEAST	333	37	Glucan 1,3-beta-glucosidase
EF1A_YEAST	296	305	Elongation factor 1-alpha
HSP75_YEAST	223	60	Heat shock protein SSB1
EF2_YEAST	239	128	Elongation factor 2
PLB1_YEAST	228	321	Lysophospholipase 1
BLH1_YEAS1	223	70	Cysteine proteinase 1, mitochondrial
FKS2_YEAST	208	30	1,3-beta-glucan synthase component GSC2
FKS1_YEAST	147	25	1,3-beta-glucan synthase component FKS1
MET17_YEAST	207	350	Protein MET17
EF3A_YEAST	203	67	Elongation factor 3A
FBRL_YEAST	197	527	rRNA 2'-O-methyltransferase fibrillarin
GAS3_YEAST	197	230	Probable 1,3-beta-glucanosyltransferase GAS3
ACT_YEAST	181	27	Actin
GAS5_YEAST	174	360	1,3-beta-glucanosyltransferase GAS5
NUM1_YEAST	161	71	Nuclear migration protein NUM1
SRP40_YEAST	152	133	Suppressor protein SRP40
DNPEP_YEAST	146	1025	Aspartyl aminopeptidase 4
SIS1_YEAST	143	not found	Protein SIS1
YJR1_YEAST	140	348	Cell wall protein YJL171C
YGP1_YEAST	111	344	Protein YGP1
CYC1_YEAST	109	209	Cytochrome c iso-1
DLDH_YEAST	103	44	Dihydrolipoyl dehydrogenase, mitochondrial
DED1_YEAS7	103	39	ATP-dependent RNA helicase DED1
SPT16_YEAST	94	38	FACT complex subunit SPT16
EXG2_YEAST	91	382	Glucan 1,3-beta-glucosidase 2
DHE4_YEAST	86	61	NADP-specific glutamate dehydrogenase 1
ENG1_YEAST	83	36	Endo-1,3(4)-beta-glucanase 1
PDC1_YEAST	82	47	Pyruvate decarboxylase isozyme 1
ENO2_YEAST	80	117	Enolase 2
G6PI_YEAST	78	27	Glucose-6-phosphate isomerase
YPS1_YEAST	74	87	Aspartic proteinase 3
RAX2_YEAST	70	46	Bud site selection protein RAX2
MID2_YEAST	67	87	Cell wall integrity sensor MID2
SWI1_YEAST	65	not found	SWI/SNF chromatin-remodeling complex subunit SWI1
ALF_YEAST	63	403	Fructose-bisphosphate aldolase
MIT1_YEAST	61	not found	Transcriptional regulator MIT1
