# Supplementary material for: Interaction of Prions Causes Heritable Traits in Saccharomyces cerevisiae
Source: PLoS Genet. 2016 Dec 27;12(12):e1006504. doi: 10.1371/journal.pgen.1006504 (PMC5189945; doi:10.1371/journal.pgen.1006504)
Supplement: S2 Table — (PDF) [file pgen.1006504.s011.pdf]

S2 Table. Oligonucleotides used in this study

Oligonucleotide	Sequence 5’-3’
ACT1F	TAACGGTTCTGGTATGTGTAAAG
ACT1R	TCATCACCAACGTAGGAGTCTT
SUP45F	AAGCATGGTAGAGGTGGTCAAT
SUP45R	TAGCCAAATCGGTCTTAAAGTCA
ACT1probe	(FAM*)CGTGCTGTCTTCCCATCTATCGTCGGT(BHQ1**)
SUP45probe	(R6G***)TGTGAGAAAGGTCGCCGAAGTTGCTGT(BHQ1)
RSWI1delta	GTTGCTGCTGCTGCTGCTGCTGTAGAGCCTGAAAACGTTGCTGCT
RSWI1deltaKanMX4	GAGCCTGAAAACGTTGCTGCTATCGATGAATTCGAGCTCG
FSWI1delta	CCATCTCTGCTTTGGCATTTCGCGTTGTTTCCTCTCACGGATTGC
FSWI1deltaKanMX4	TTTCCTCTCACGGATTGCCGTACGCTGCAGGTCGAC
FSWI1deltach	CAGATTATTGTTCACCAGGT
RSWI1deltach	TGTTCGGAGTAGAAGATGA
RRNQ1deltaKanMX4	GTTGAGAAAAGTTGCCAGAATCGATGAATTCGAGCTCG
FRNQ1deltaKanMX4	GTCTCATTTTTCTCAAGGACGTACGCTGCAGGTCGAC
FRNQ1delta	ATGGATACGGATAAGTTAATCTCAGAGGCTGAGTCTCATTTTTCTCAAGGA
RRNQ1delta	TCAGTAGCGGTTCTGGTTGCCGTTATTGTTCTGTTGAGAAAAGTTGCCAGA
FRNQ1deltach	AGCCACAGTGATGACACACT
RRNQ1deltach	AGGTAGGAACTTGCCATGGA
MIT1F	CGCGGATCCATGGATATCGAGCCTACT
MIT1R	CGCCCGCGGTTGTGTAGTAGTTGAAGTGTT
MIT1_prom_F	CCGATCGATGCTTCGATTGGTAACAGTG
MIT1_prom_R	GCCGGATCCTTAGAAATTTACGTTGTCCTTG
FMIT1deltach	TCAGAGCAAGACACCTCCA
RMIT1deltach	CGTTCCCTGCATTAGTATTG
FCFPSacII	GTCCCCGCGGAGTAAAGGAGAACTTTTC
RCFPSacI	TCCGAGCTCTCATTTGTATAGTTCATCCATGCC
FSWI1(1)HindIII	GACTCGAAGCTTTATATGGATTTCTTTAATTTGAATAAT
RSWI1(889)BamHI	GATGAATGGATCCTTTATTATTGCTGTTATTACG

*FAM, FAM fluorophore
**BHQ1, BHQ1 quencher of fluorescence
***R6G, R6G fluorophore
